# Supplementary material for: Genomic consequences of residual recombination in a hybrid apomictic hickory complex
Source: Nat Commun. 2026 Feb 5;17:2376. doi: 10.1038/s41467-026-68867-6 (PMC12982824; doi:10.1038/s41467-026-68867-6)
Supplement: Supplementary file 1 — Supplementary Information [file 41467_2026_68867_MOESM1_ESM.pdf]

Supplementary Information for
"Genomic consequences of residual recombination in a hybrid apomictic hickory complex"

Supplementary Method 1. Karyotype Analysis for *Carya hunanensis*

To determine the karyotype and ploidy level of the hybrid species *Carya hunanensis*, we selected the JZ morphotype as a representative. Chromosome preparation was conducted using root tip meristem tissue from germinated seeds after one week of growth. Chromosome painting techniques followed [1] and [2] with adaptations performed for root tissues. The dispersed cells of chromosome spread at meiotic stages were counterstained with DAPI (4′,6-diamidino-2-phenylindole) and fluorescence in situ hybridization (FISH) with telomere repeats Oligo-$(TTTAGGG)_6$ as probes to determine the chromosome number. In addition, we used 5S rDNA and 18S rDNA repeats as probes for fluorescence in situ hybridization to determine chromosome ploidy. The chromosome number and ploidy were photographed under an Olympus BX63 fluorescence microscope.

DAPI staining revealed a chromosome count of 32, with chromosome lengths ranging from 1.0 to 2.0 μm, predominantly metacentric and telocentric chromosomes, indicating a relatively small genome size. FISH analysis detected distinct hybridization signals: one strong and one weak red signal on two chromosomes for 5S rDNA, and strong green signals on two chromosomes for 18S rDNA. These results confirm that *C. hunanensis* is diploid, with a karyotype of 2n = 2x = 32 (Supplementary Fig. 2), consistent with previous reports for other *Carya* species [3, 4]. This karyotypic characterization supports subsequent population genomic analyses.

Supplementary Method 2. Plant materials, genome sequencing, assembly and and haplotype phasing

Fresh young leaves of *C. hunanensis* were collected from an adult tree in Tongdao County, Hunan Province, China (26°6′53.63″N, 109°41′39.61″E) and immediately stored in liquid nitrogen. Genomic sequencing was conducted by BioMarker (Beijing, China). For short-read sequencing, DNA libraries with 350 bp insert sizes were constructed and sequenced using the Illumina NovaSeq 6000 platform. A total of 31.27 Gb (~44.57× coverage) of data were obtained, and raw reads were quality-filtered using Trimmomatic v0.36 [5] with default parameters. Approximately 10 μg of sheared DNA was subjected to size selection (CLR: ~20 kb; CCS: ~15 kb) using the Megaruptor® 2 system. SMRT bell libraries were prepared following the manufacturer's instructions (Pacific Biosciences, CA, USA) and sequenced on a PacBio Sequel II platform using circular consensus sequencing (CCS) technology. After filtering low-quality reads and adapter sequences, 25.07 Gb (~18.48× coverage) of clean subreads were obtained. A Hi-C library with an insert size of 300–700 bp was constructed following the protocol of Xie, Zheng [6]. Sequencing on the Illumina NovaSeq 6000 platform produced 96.93 Gb (~71.44× coverage) of Hi-C data. Invalid read pairs, including dangling-end, self-cycle, re-ligation, and dumped products, were filtered using HiC-Pro v2.8.1 [7].

Genome size, heterozygosity, and repeat content were estimated using Illumina clean reads. K-mer (19-mer) analysis was conducted with Jellyfish v2.1.4 [8], and genome characteristics were assessed using GenomeScope v2.0 [9]. The estimated genome size of *C. hunanensis* was 701.66 Mb, with a heterozygosity rate of 2.99% and a repeat content of 54.34%. De novo genome assembly was performed using hifiasm v0.16 [10], producing a total contig length of 1,356,769,403 bp, and a contig N50 of 11,316,232 bp. For chromosome-level scaffolding, an initial preassembly step was conducted to correct errors by segmenting scaffolds into ~50 kb fragments. Clean Hi-C read pairs were aligned to the assembled contigs using BWA-MEM [11], retaining only uniquely mapped reads. Scaffolding was performed using LACHESIS [12] with the following parameters: CLUSTER_MIN_RE_SITES = 100, CLUSTER_MAX_LINK_DENSITY = 2, ORDER_MIN_N_RES_IN_TRUNK = 154, and ORDER_MIN_N_RES_IN_SHREDS = 158. Misplaced or misoriented scaffolds showing inconsistent chromatin interaction patterns were manually corrected. In total, 1,344,630,262 bp (99.11% of the assembled genome) were anchored to 32 chromosomes. Among these, 1,306,056,218 bp (97.13% of the anchored sequences) were assigned with a defined order and orientation.

The initial assembly obtained was twice the size of the anticipated genome size, suggesting that it contained two haplotypes. Since *C. hunanensis* is thought to be a hybrid between *C. tonkinensis* and the ancestor of *C. cathayensis* and *C. dabieshanensis* [13], we separated these haplotypes using a method based on individual genome coverage. Illumina resequencing reads from five representative individuals of *C. cathayensis*, *C. dabieshanensi*, the JZ and YL morphotypes of *C. hunanensis*, and *C. tonkinensis* were aligned to the 16 pseudochromosome pairs. The highest coverage from *C. cathayensis* and *C. dabieshanensis* was used to define homologous chromosomes as ChrA, with

regions of lower coverage designated as ChrB (opposite coverage patterns were observed in *C. tonkinensis*, Supplementary Fig. 6). To validate the accuracy of haplotype phasing, haplotype-specific repetitive DNA sequences were identified using SubPhaser [14] with default parameters. This analysis confirmed the haplotypes identified by the genome coverage-based method. Consequently, the *C. hunanensis* genome was divided into two haplotypes, referred to as *C. hunanensis* E (East) and *C. hunanensis* W (West) (Fig. 1), reflecting their inferred parental geographic origins.

The completeness of the genome assembly was evaluated by mapping Illumina whole-genome sequencing reads back to the assembly using BWA v0.7.12 [15] and by Benchmarking Universal Single-Copy Orthologs (BUSCO) v5.2.2 [16], utilizing the "embryophyte_odb10" database (Supplementary Fig. 5).

Supplementary Method 3. Annotation of genes, repetitive elements, and non-coding RNAs

Gene Prediction and Functional Annotation

To annotate the genomes of *C. hunanensis*, a combination of *ab initio* prediction, homology-based inference, and transcriptome data from RNA sequencing (RNA-Seq) was employed. For ab initio prediction, gene models were generated using AUGUSTUS v3.3.2 [17] and SNAP v.2013-11-2 [18]. For the homology-based approach, GeMoMa v1.7 [19] was used with reference gene models from *Arabidopsis thaliana*, *Glycine max*, *Juglans regia*, and *C. illinoinensis*. For the transcript-based prediction, RNA-Seq data from mixed tissues (leaf, bud, and fruit) of the same plant were aligned to the reference genome using HISAT v2.0.4 [20] and assembled with StringTie v1.2.3 [21]. Gene predictions based on these assembled transcripts were carried out using GeneMarkS-T v5.1 [22]. Additionally, PASA v2.0.2 [23] was employed to predict genes using unigenes and full-length transcripts obtained from PacBio sequencing and assembled with Trinity v2.11 [24]. Gene models from all approaches were integrated using the EvidenceModeler (EVM) software v1.1.1 and subsequently refined with PASA v2.3.3 [25]. The final gene models were annotated by searching against multiple databases, including GenBank Non-Redundant (NR, 20200921), TrEMBL (202005), Pfam (33.1), SwissProt (202005), eukaryotic orthologous groups (KOG, 20110125), Gene Ontology (GO, 20200615), and Kyoto Encyclopedia of Genes and Genomes (KEGG, 20191220).

Repetitive Element Annotation

Repetitive sequences, including transposable elements (TEs) and tandem repeats, were identified using a combination of homology-based and de novo approaches. First, a de novo repeat library was constructed using RepeatModeler [26], which integrates RECON v1.08 [27] and RepeatScout [28] for repeat identification. Full-length long terminal repeat retrotransposons (fl-LTR-RTs) were detected using LTRharvest v1.5.9 [29] and LTR_finder v2.8 [30], and a high-quality, non-redundant LTR library was curated with LTR_retriever [31]. A species-specific TE library was generated by integrating the de novo TE sequences with the known Dfam v3.2 database. The final TE sequences in the *Carya hunanensis* genome were identified and classified through homology-based searches using RepeatMasker v4.10[32]. Tandem repeats were annotated using Tandem Repeats Finder (TRF 409) and the MIcroSAtellite Identification Tool (MISA v2.1).

Pseudogene Prediction

Pseudogenes, which share sequence similarity with functional genes but have lost their biological function due to mutations, were identified through a homology-based approach. First, GenBlastA v1.0.4 [33] was used to scan the genome after masking functional gene regions. Candidate pseudogenes were further analyzed for premature stop codons and frameshift mutations using GeneWise v2.4.1 [34].

Non-Coding RNA Annotation

Non-coding RNAs (ncRNAs), including tRNAs, rRNAs, miRNAs, snoRNAs, and snRNAs, were identified using specialized tools. tRNAs were predicted with tRNAscan-SE v2.0.7 [35] using eukaryote-specific parameters. Ribosomal RNA (rRNA) genes were identified with barrnap v0.9. miRNAs were annotated by searching against the miRBase (release 21) database [36]. Small nucleolar RNAs (snoRNAs) and small nuclear RNAs (snRNAs) were predicted using Infernal v1.1 [37] against the Rfam v14.5 database [38] under default parameters.

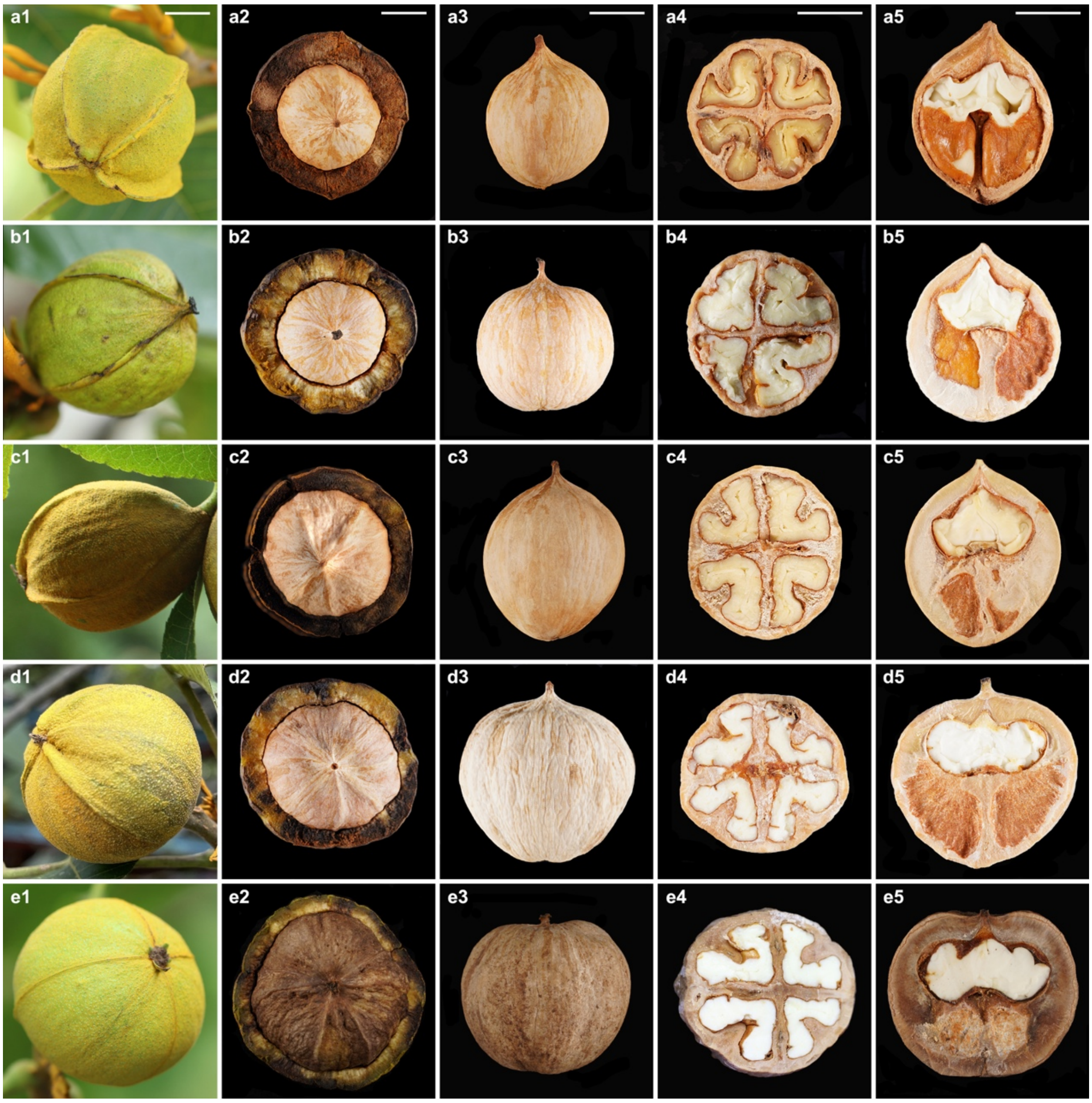

Supplementary Figure 1. Fruit morphological characteristics of five hickory taxa. **a** *Carya cathayensis*; **b** *C. dabieshanensis*; **c** TD morphotype of *C. hunanensis*; **d** YL morphotype of *C. hunanensis*; **e** *C. tonkinensis*. **1** Husk; **2** Shell, vertical view; **3** Shell, lateral view; **4** Transversal section of nuts, showing 4 chambers; **5** Longitudinal section of nuts. The TD and YL morphotypes of *C. hunanensis* are named after their respective distribution centers in Tongdao County and Yuanling County, Hunan Province, China. White scale bars indicate 1 cm, with the same scale size for each column.

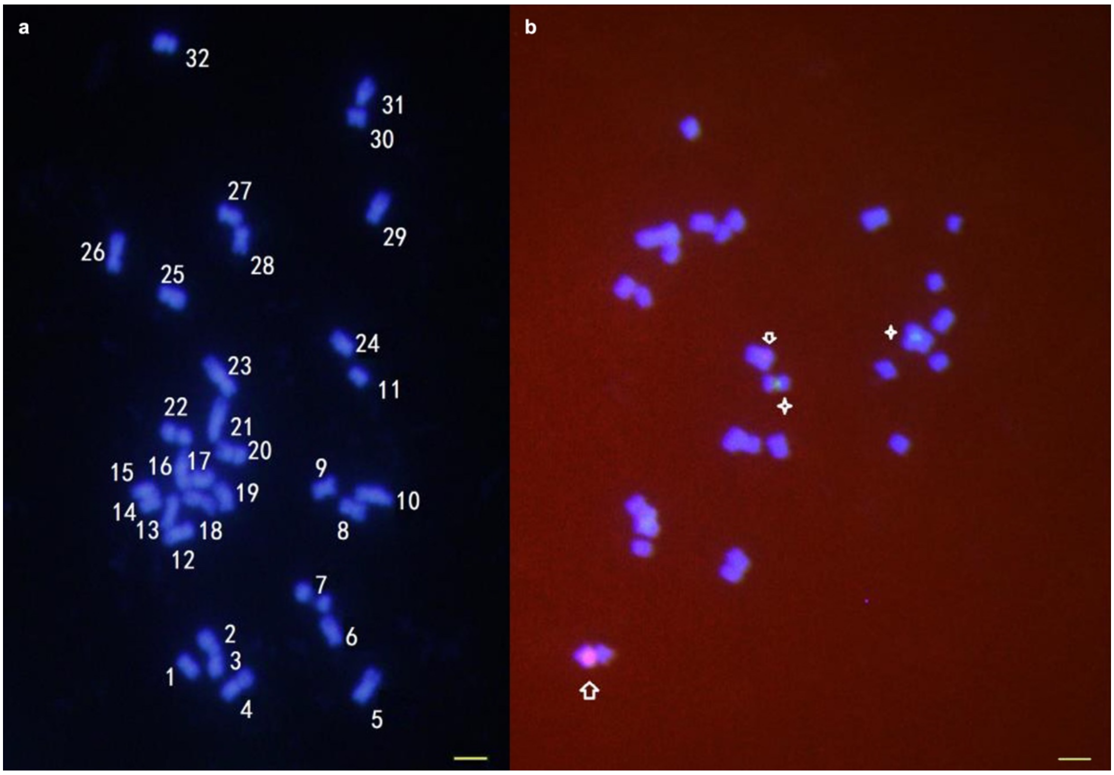

Supplementary Figure 2. FISH results confirm that *Carya hunanensis* is diploid with a karyotype of 2n = 2x = 32.

a DAPI fluorescence staining reveals that *C. hunanensis* has 32 chromosomes, ranging in length from 10 to 20 µm, primarily comprising submetacentric and telocentric chromosomes, with a relatively small genome. **b** Ploidy-estimating of chromosomes within the *C. hunanensis* karyotype using FISH with 5SrDNA sequence (red) and 18SrDNA sequence (green) as the probes. Scale bar, 5 µm. Three independent biological replicates. The analyzed material was collected from the TD morphotype of *C. hunanensis*.

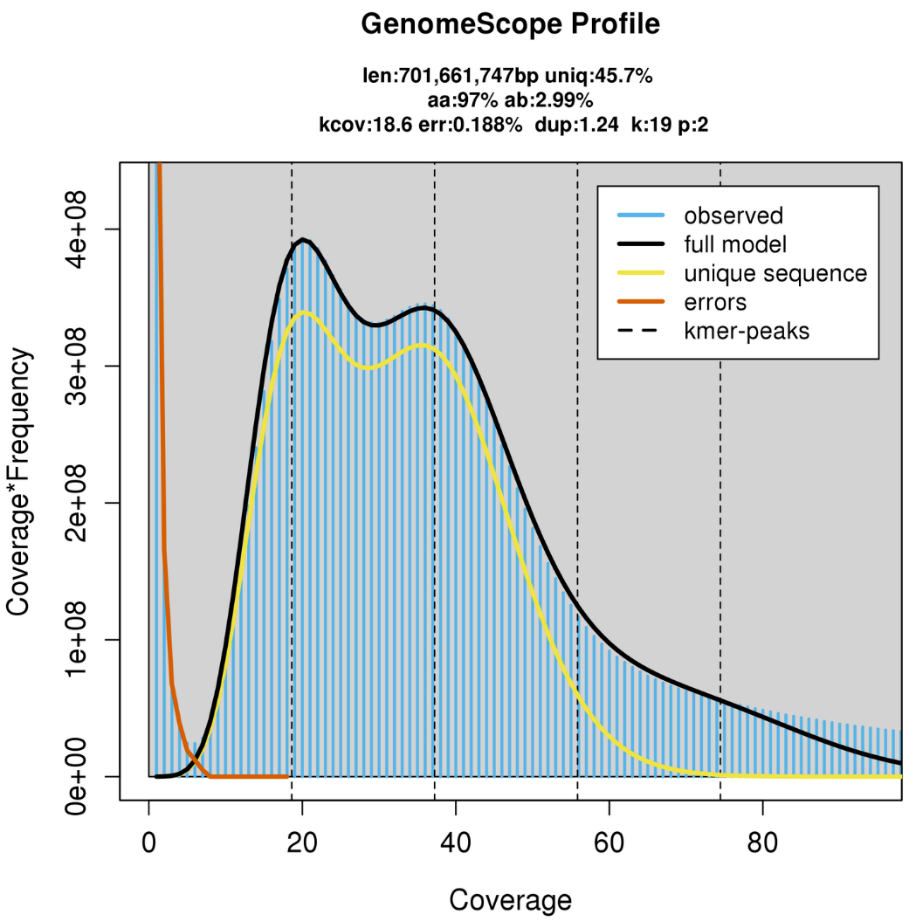

Supplementary Figure 3. GenomeScope plots for heterozygous species of *Carya hunanensis*.

The genome size of *C. hunanensis* was estimated to be ~701.66 Mb with a heterozygosity of 2.99% and a repetitive content of 54.34%.

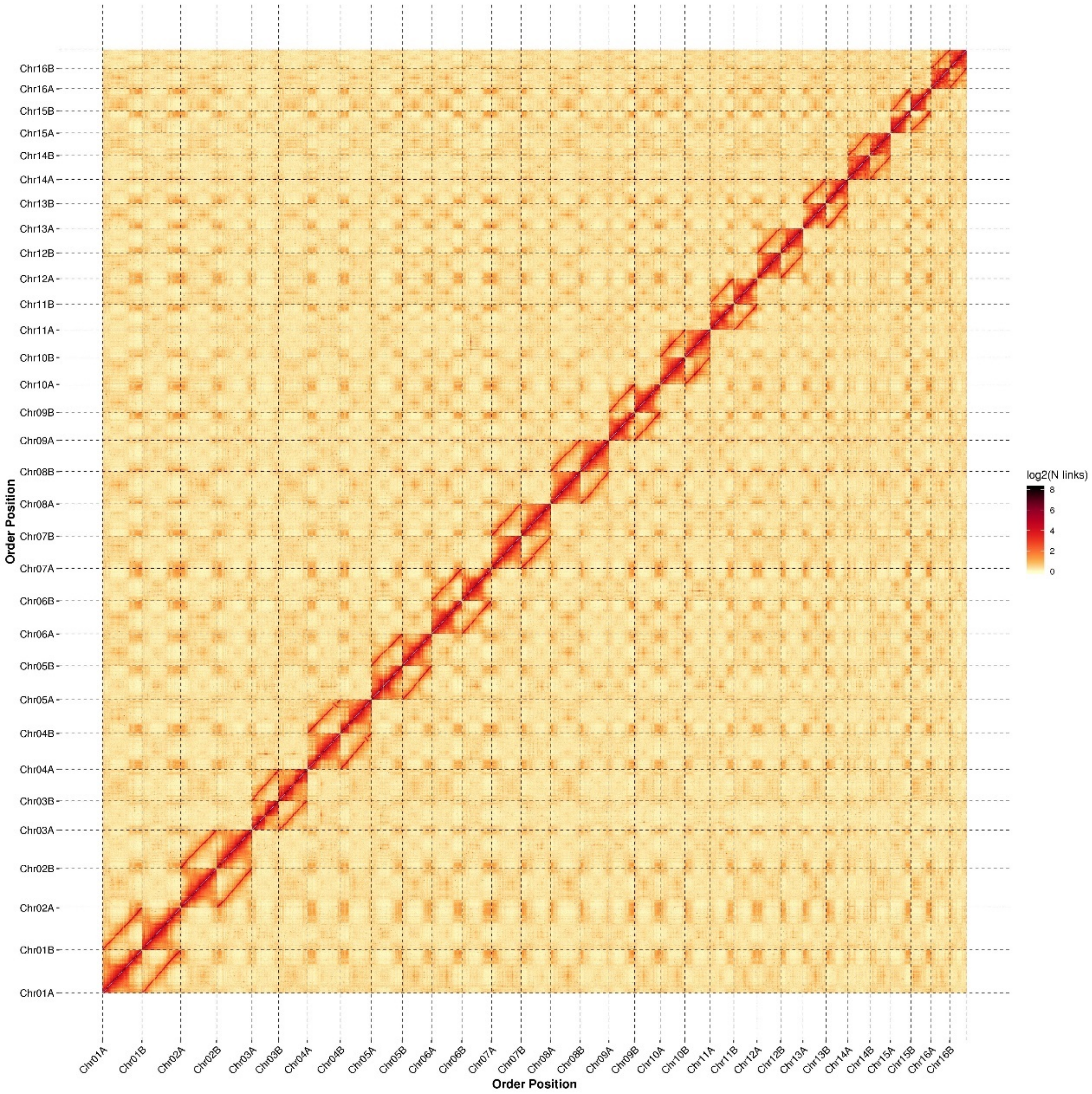

Supplementary Figure 4. Hi-C intrachromosomal contact map for 16 chromosome pairs of *Carya hunanensis*.

The intensity of the pixels reflects the number of Hi-C links between 500-kb windows on chromosomes, presented on a logarithmic scale, with darker red indicating higher contact probability.

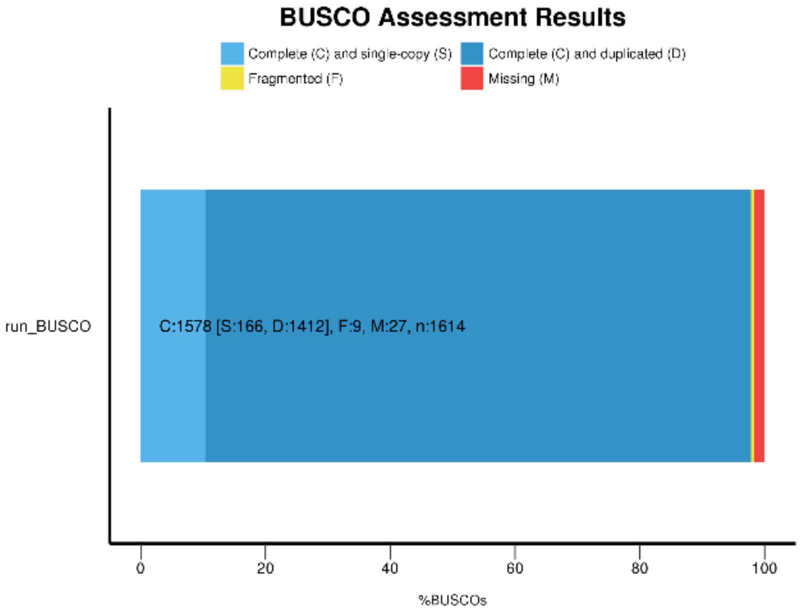

Supplementary Figure 5. Genome completeness of *Carya hunanensis* evaluated using Benchmarking Universal Single-Copy Orthologs (BUSCO) based on 1,614 conserved single-copy orthologous groups in plants.

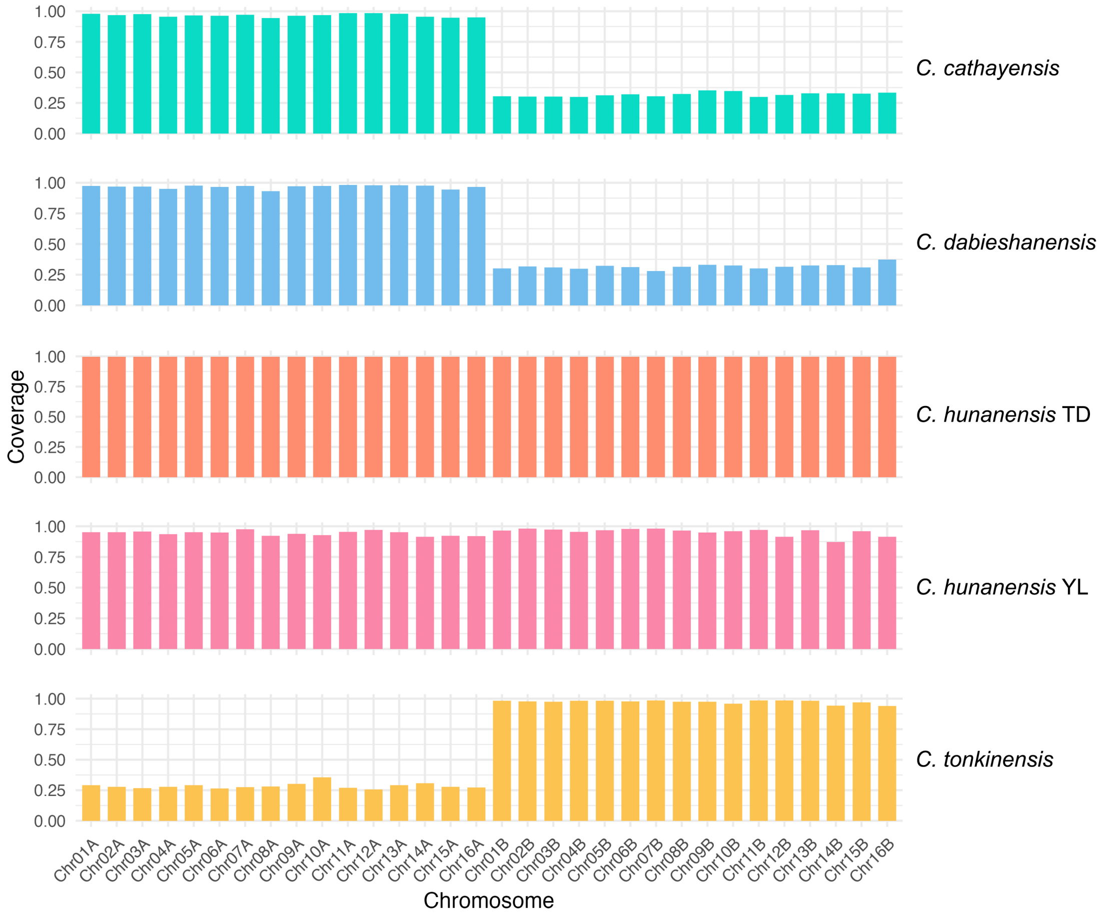

Supplementary Figure 6. Genome coverage of representative individuals from five hickory taxa mapped to the 16 chromosome pairs of *Carya hunanensis*.
Two distinct morphotypes (TD and YL) were analyzed in *C. hunanensis*.

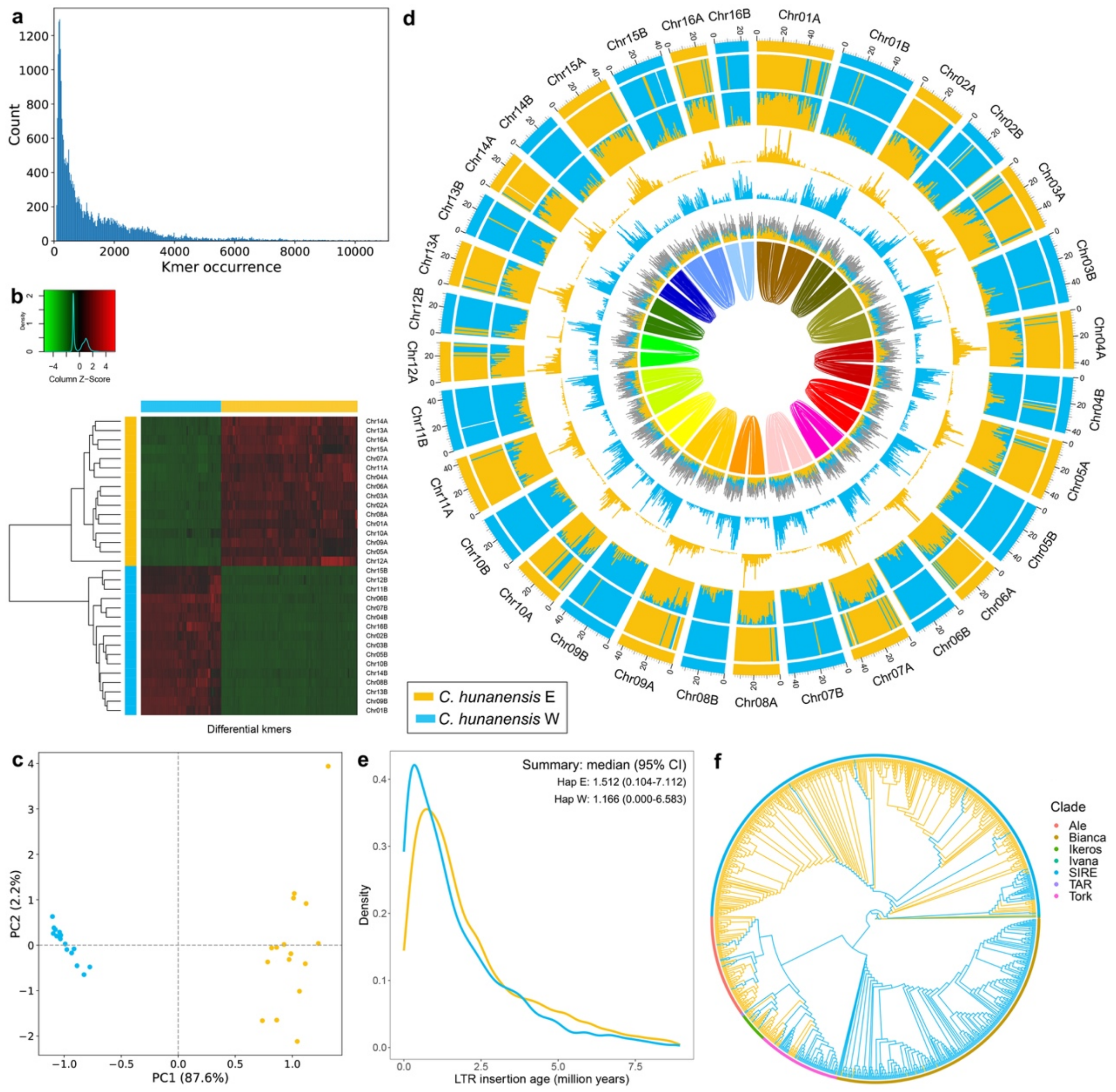

Supplementary Figure 7. Haplotype genome phasing and characterization of *Carya hunanensis*.

a The histogram of differential k-mers among homoeologous chromosome sets. **b** Heatmap and clustering of differential k-mers. The x-axis, differential k-mers; y-axis, chromosomes. The vertical color bar, each chromosome is assigned to which haplotype; the horizontal color bar, each k-mer is specific to which haplotype (blank for non-specific kmers). **c** Principal component analysis (PCA) of differential k-mers. Points indicate chromosomes. **d** Chromosomal characteristics (window size: 1 Mb). Rings from outer to inner: (**1**) haplotype assignments by a k-Means algorithm. (**2**) Significant enrichment of haplotype-specific k-mers (blank for non-enriched windows). (**3**) Normalized proportion of haplotype-specific k-mers. (**4-6**) Density distribution (count) of each haplotype-specific k-mer set. (**7**) Density distribution (count) of haplotype-specific LTR-RTs and other LTR-RTs (the most outer, in grey color). (**8**) Homoeologous blocks of each homoeologous chromosome set. **e** Insertion time of haplotype-specific LTR-RTs. **f** A phylogenetic tree of 1,000 randomly subsampled LTR/Copia elements.

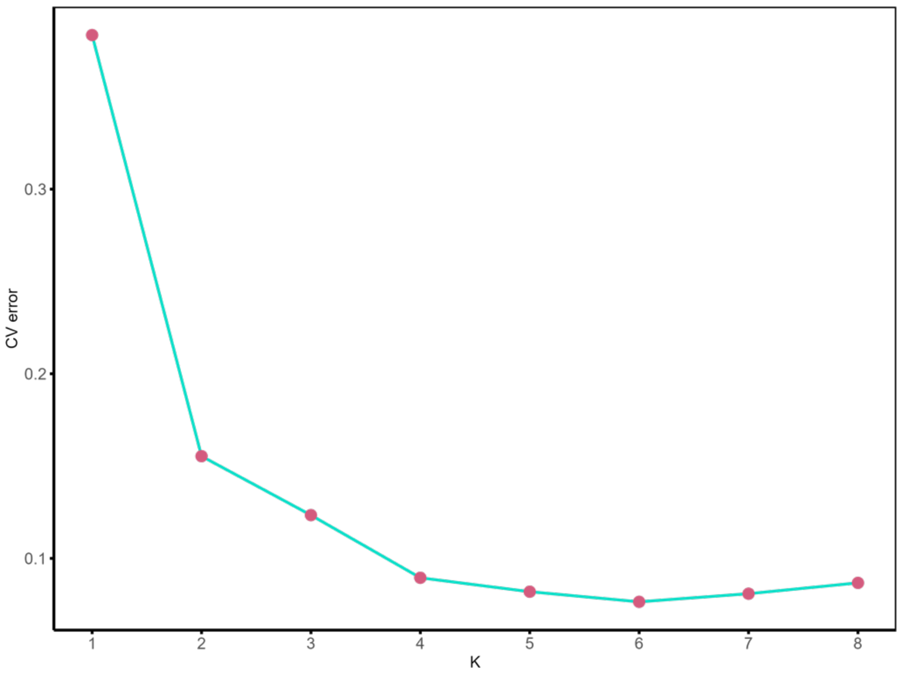

Supplementary Figure 8. Cross-validation (CV) errors of ADMIXTURE analysis based on SNP data from 195 adult individuals of four hickory species.

Means of CV errors were calculated based on *K* values ranging 1 to 8 with 20 independent runs.

C. cathayensis	0.0060	0.0138	0.0145	0.0253
0.3151	***C. dabieshanensis***	0.0137	0.0145	0.0252
0.4767	0.4280	***C. hunanensis* TD**	0.0145	0.0175
0.5904	0.5203	0.1931	***C. hunanensis* YL**	0.0180
0.7615	0.7289	0.4223	0.4408	***C. tonkinensis***

Supplementary Figure 9. Matrix of relative genetic divergence for pairwise comparisons of five hickory taxa.

The F_{ST} values are shown in the lower-left corner of the matrix, and the d_{xy} values are shown in the upper-right corner. Both morphotypes, TD and YL, of *Carya hunanensis* are included.

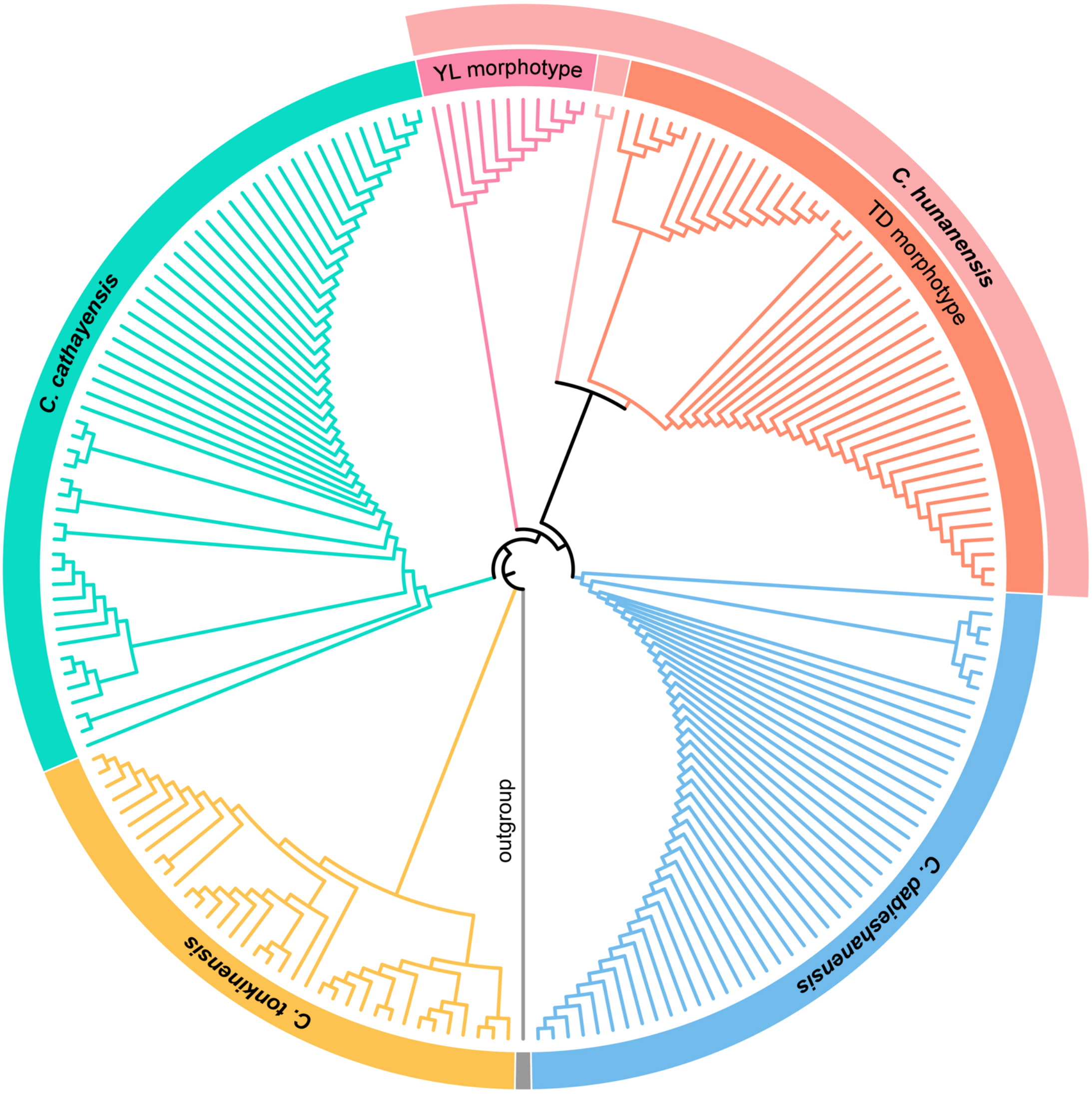

Supplementary Figure 10. Phylogenetic tree of five hickory taxa constructed from chloroplast genomes.

The maximum likelihood phylogenetic tree includes 195 individuals from five *Carya* taxa (*C. cathayensis*, *C. dabieshanensis*, the TD morphotype of *C. hunanensis*, the YL morphotype of *C. hunanensis* and *C. tonkinensis*), with one individual of *C. illinoinensis* serving as an outgroup (gray). Branch colors represent different taxa.

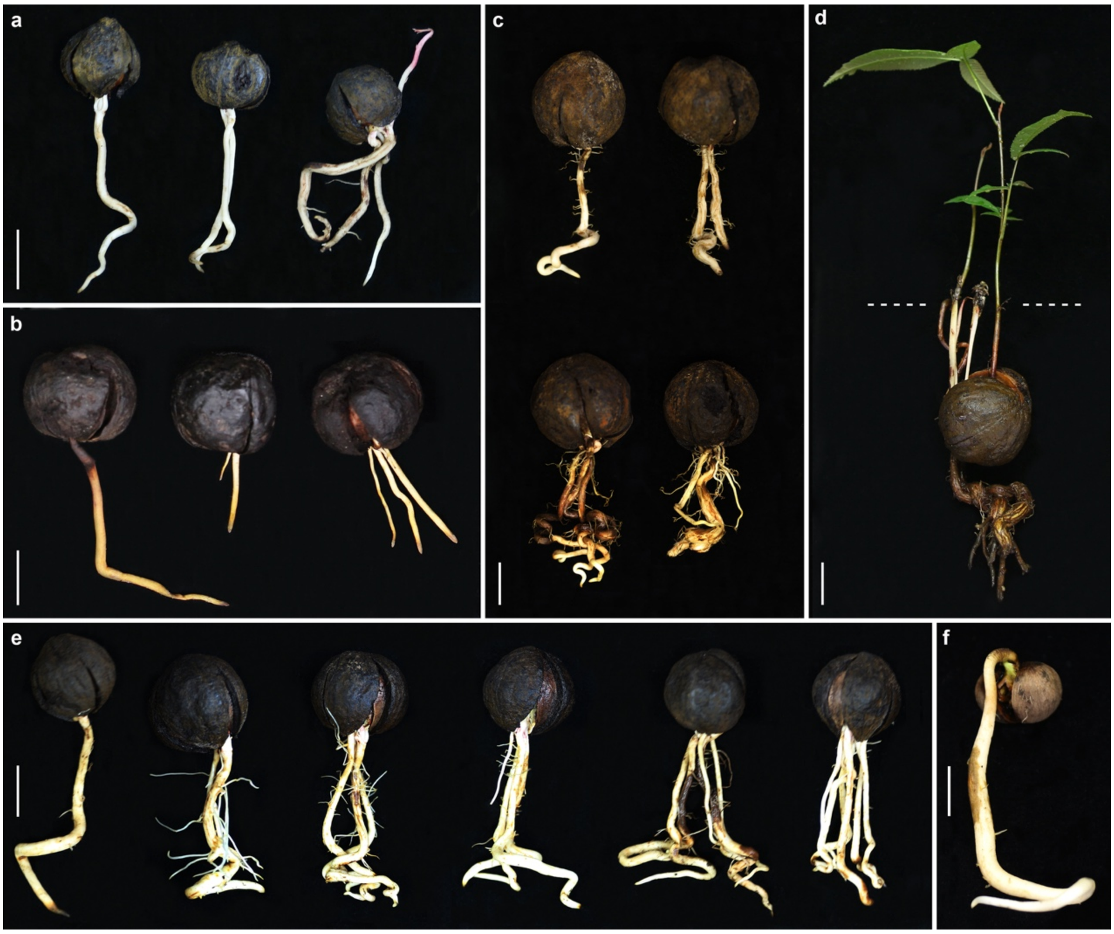

Supplementary Figure 11. Multiple seedlings per seed after germination in four hickory species.

a *Carya cathayensis* (1-3 seedlings); **b** *C. dabieshanensis* (1-3 seedlings); **c-d** YL morphotype of *C. hunanensis* (1-4 seedlings), with (D**)** showing the seedings after 2 months, where only two of the four seedlings successfully emerged, as indicated by the dashed line representing the soil surface; **e** TD morphotype of *C. hunanensis* (1-8 seedlings); **f** *C. tonkinensis* (1 seedling). Seedling numbers were observed from germination of 120 seeds per taxa. White scale bars indicate 2 cm.

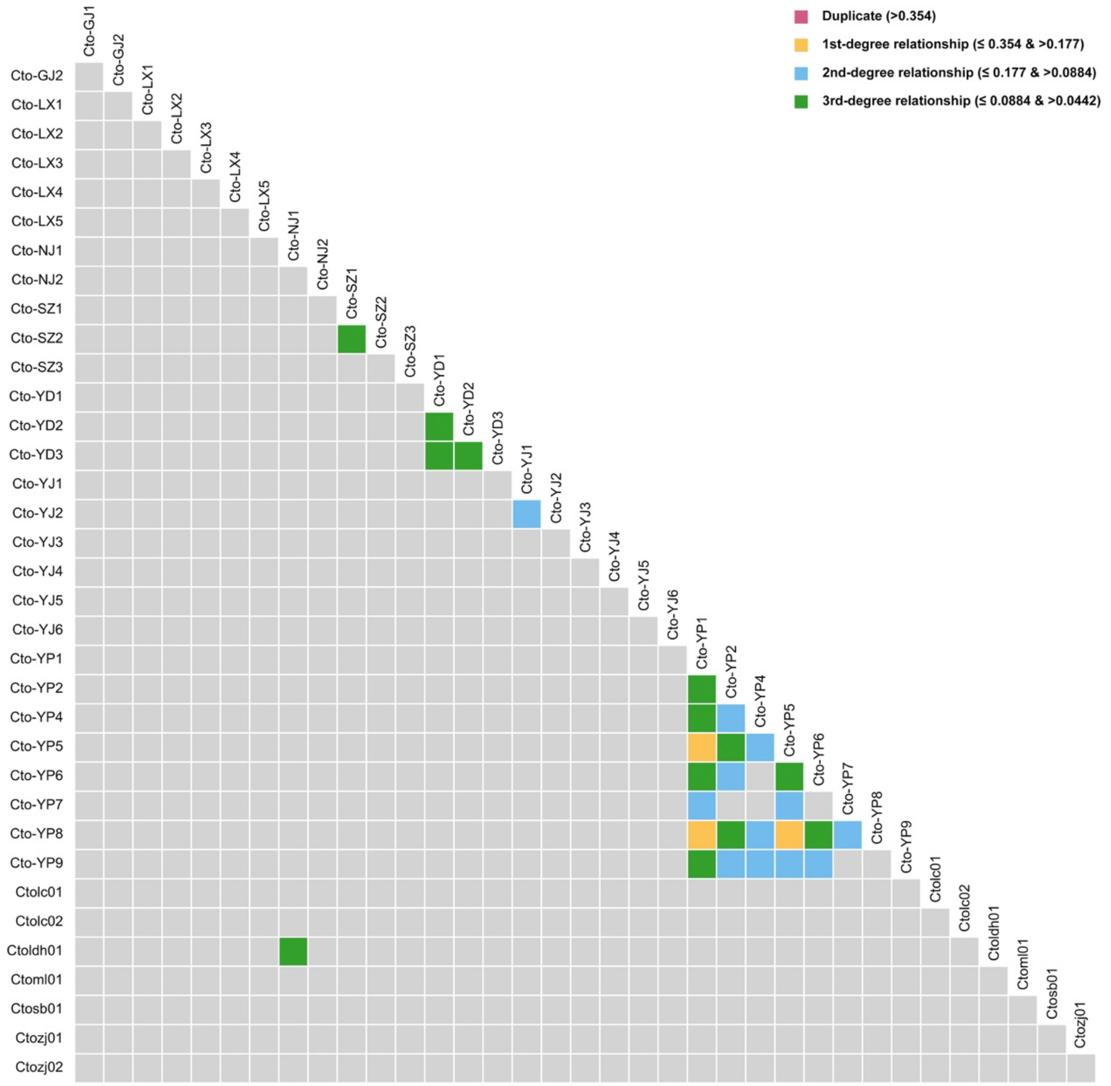

Supplementary Figure 12. Estimated genetic relatedness among 36 individuals of *Carya tonkinensis*.

Close kinship within three generations is primarily observed among individuals from the same geographic populations, including SZ (Shizong County, Yunnan Province, China), YJ (Yuanjiang, Yunnan Province, China), and YP (Yongping County, Yunnan Province, China), likely resulting from inbreeding due to geographic proximity. These kinship patterns support the conclusion that *C. tonkinensis* is a sexually reproducing species.

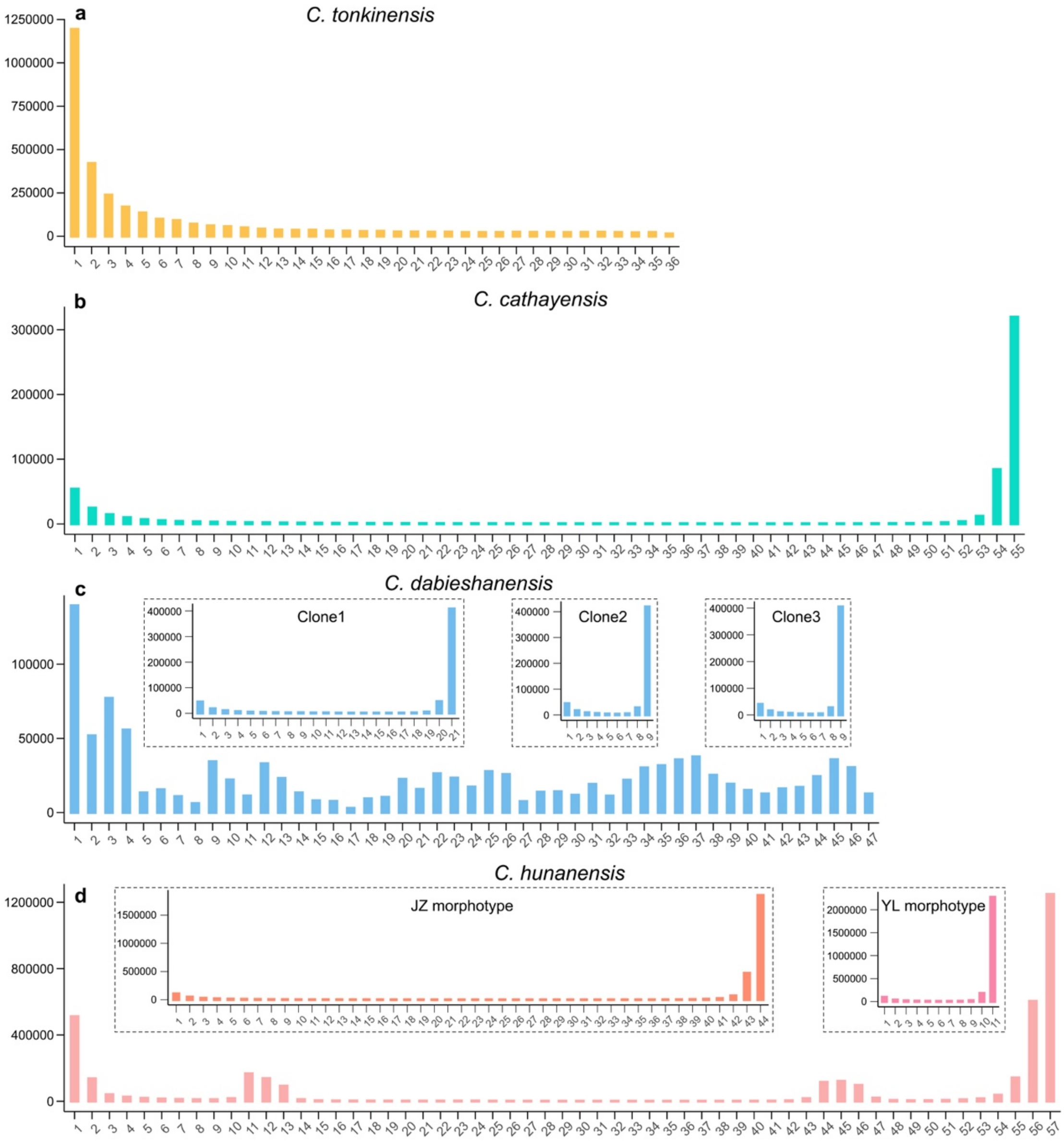

Supplementary Figure 13. Folded site frequency spectrum (SFS) of four hickory species.

a *Carya tonkinensis* exhibited a unimodal site frequency spectrum (SFS) characteristic of sexually reproducing plants, with a pronounced first-bin peak composed mostly of singletons and few (n−1)-tons. **b** The apomictic species *C. cathayensis* displayed a distinctly bimodal distribution, with a pronounced peak in the first and last bin, reflecting an excess of alleles at high minor allele frequency. **c−d** The SFS of *C. dabieshanensis* (C) *and C. hunanensis* (D) were multimodal. In particular, the three largest clonal groups of *C. dabieshanensis* (21, 9, and 9 individuals) and both TD (44 individuals) and YL (11 individuals) morphotypes of *C. hunanensis* all resembled the bimodal spectrum of *C. cathayensis*.

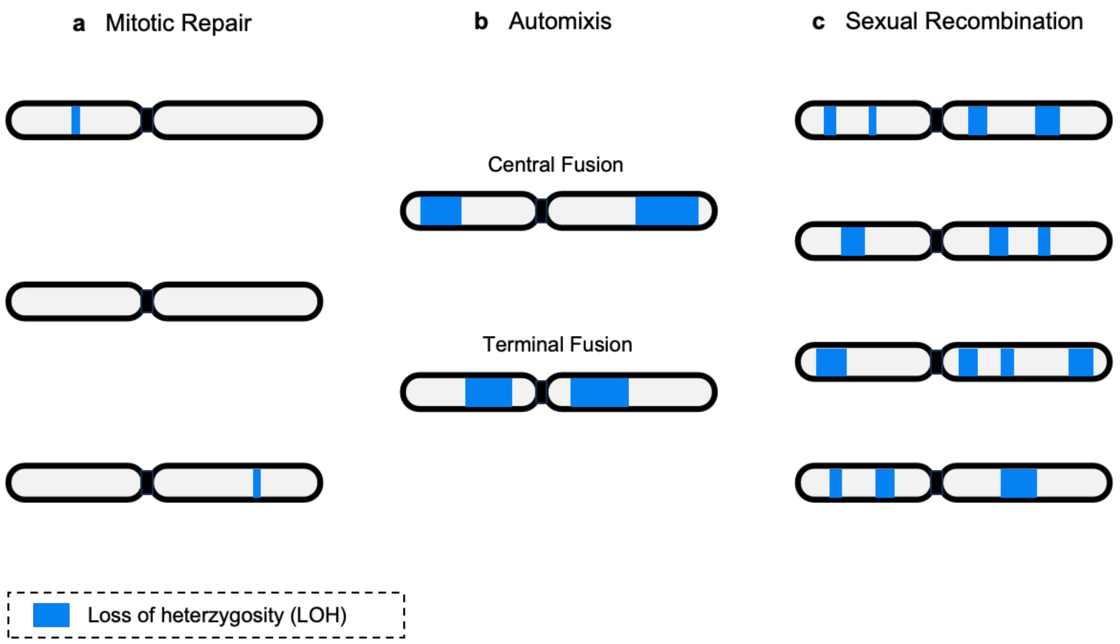

Supplementary Figure 14. Schematic comparison of loss-of-heterozygosity (LOH) signatures expected under different mechanisms.

a Mitotic repair typically produces very short, localized LOH tracts arising from gene conversion or break-induced replication, usually restricted to a few hundred bp to kb. **b** Automixis generates genome-wide LOH with structured patterns: terminal fusion yields LOH around centromeres, whereas central fusion produces LOH at chromosome ends while retaining heterozygosity near centromeres. **c** Residual sexual recombination results in stochastic LOH tracts of variable chromosomal positions across embryos, reflecting random meiotic crossing-over. Blue regions indicate LOH, grey regions indicate heterozygous segments.

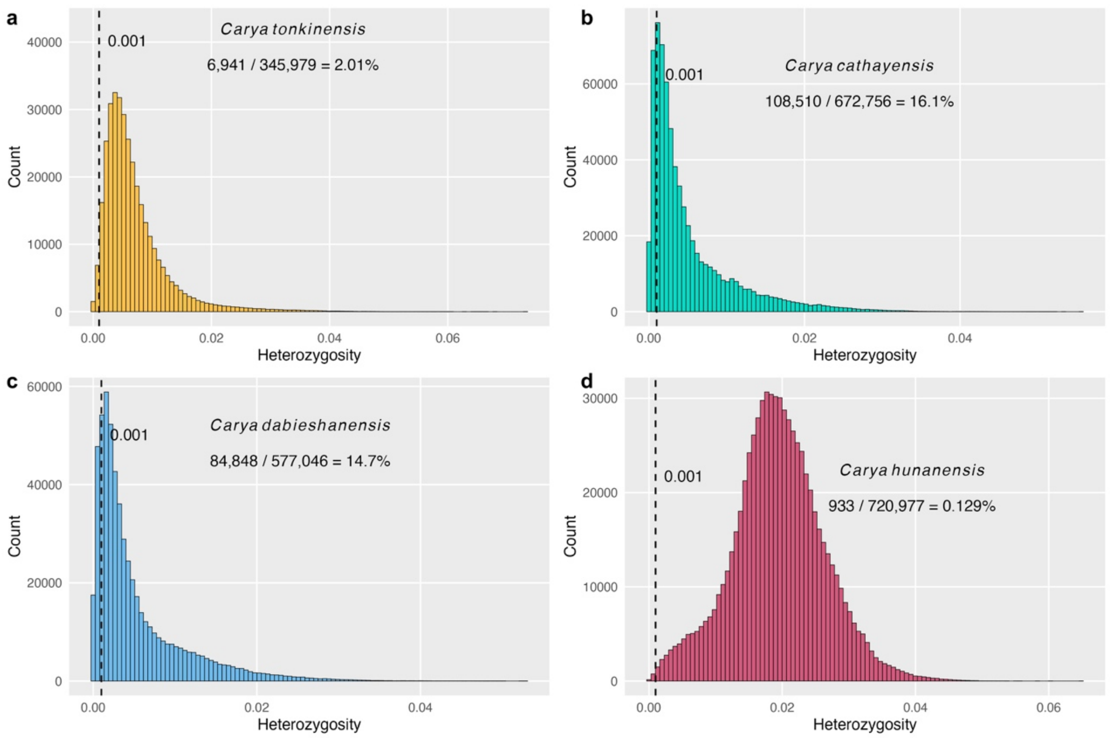

Supplementary Figure 15. Distribution of window heterozygosity across sexual control and three apomictic species.

Histograms show heterozygosity values calculated in 50-kb windows for 36 resequenced adult individuals of *C. tonkinensis* (**a**), 55 of *C. cathayensis* (**b**), 47 of *C. dabieshanensis* (**c**), and 57 of *C. hunanensis* (**d**). The dashed vertical line marks the predefined cutoff of 0.001 used to define loss of heterozygosity (LOH). In the sexual control *C. tonkinensis* (A), only 2.01% of 345,979 windows fall below this value, confirming that it represents the extreme left tail of the sexual heterozygosity distribution and provides a conservative threshold for LOH detection.

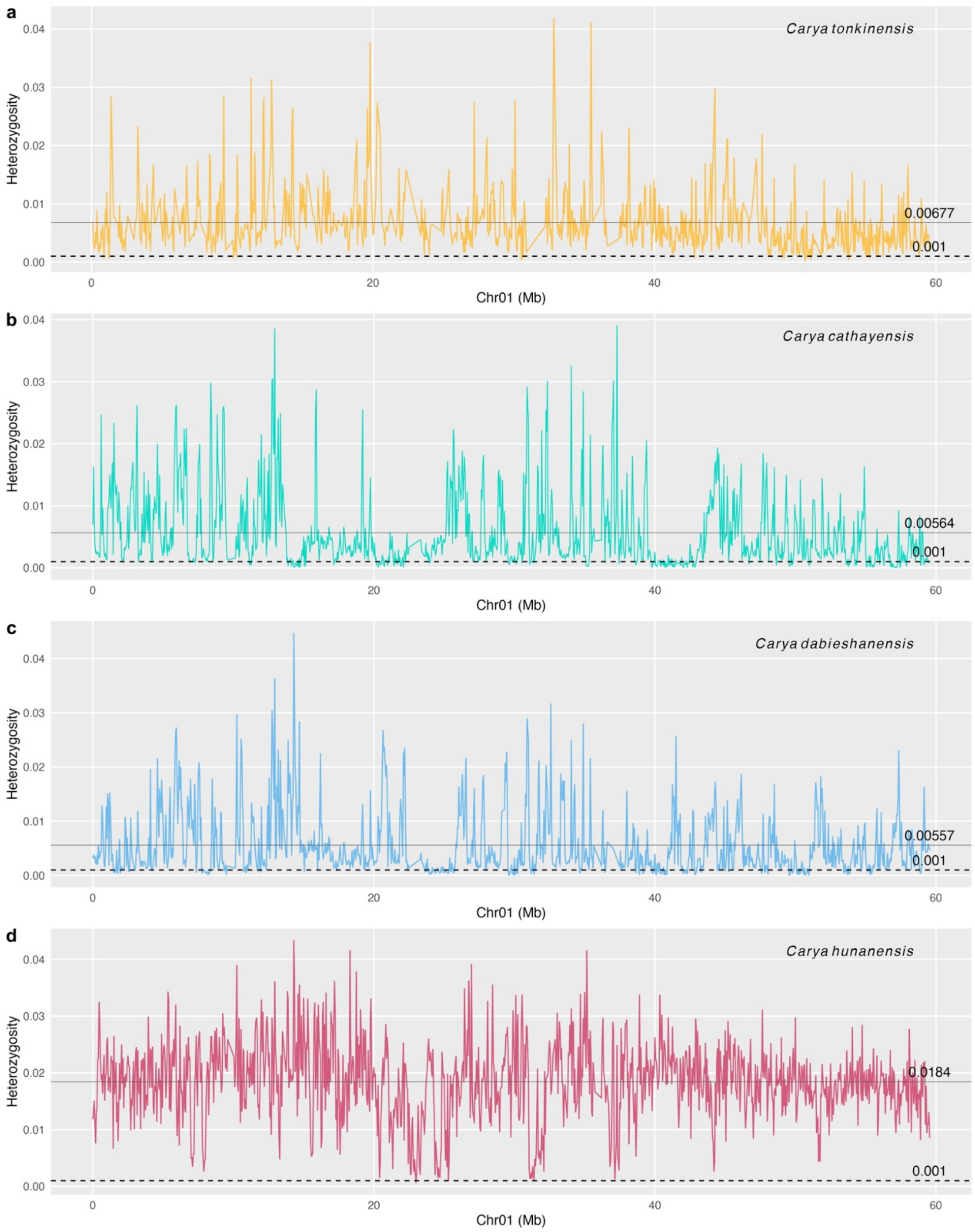

Supplementary Figure 16. Genome-wide heterozygosity plots for representative individuals of the sexual control and three apomictic species.

Heterozygosity was calculated in 50-kb windows along chromosome 01 for one adult individual of each species: *C. tonkinensis* (**a**), *C. cathayensis* (**b**), *C. dabieshanensis* (**c**), and *C. hunanensis* (**d**). The plots show a generally stable background level of heterozygosity, punctuated by rare LOH segments where heterozygosity approaches zero. The dashed black line marks the predefined cutoff of 0.001 used to define LOH, while the thin solid black line indicates the chromosome-wide average heterozygosity.

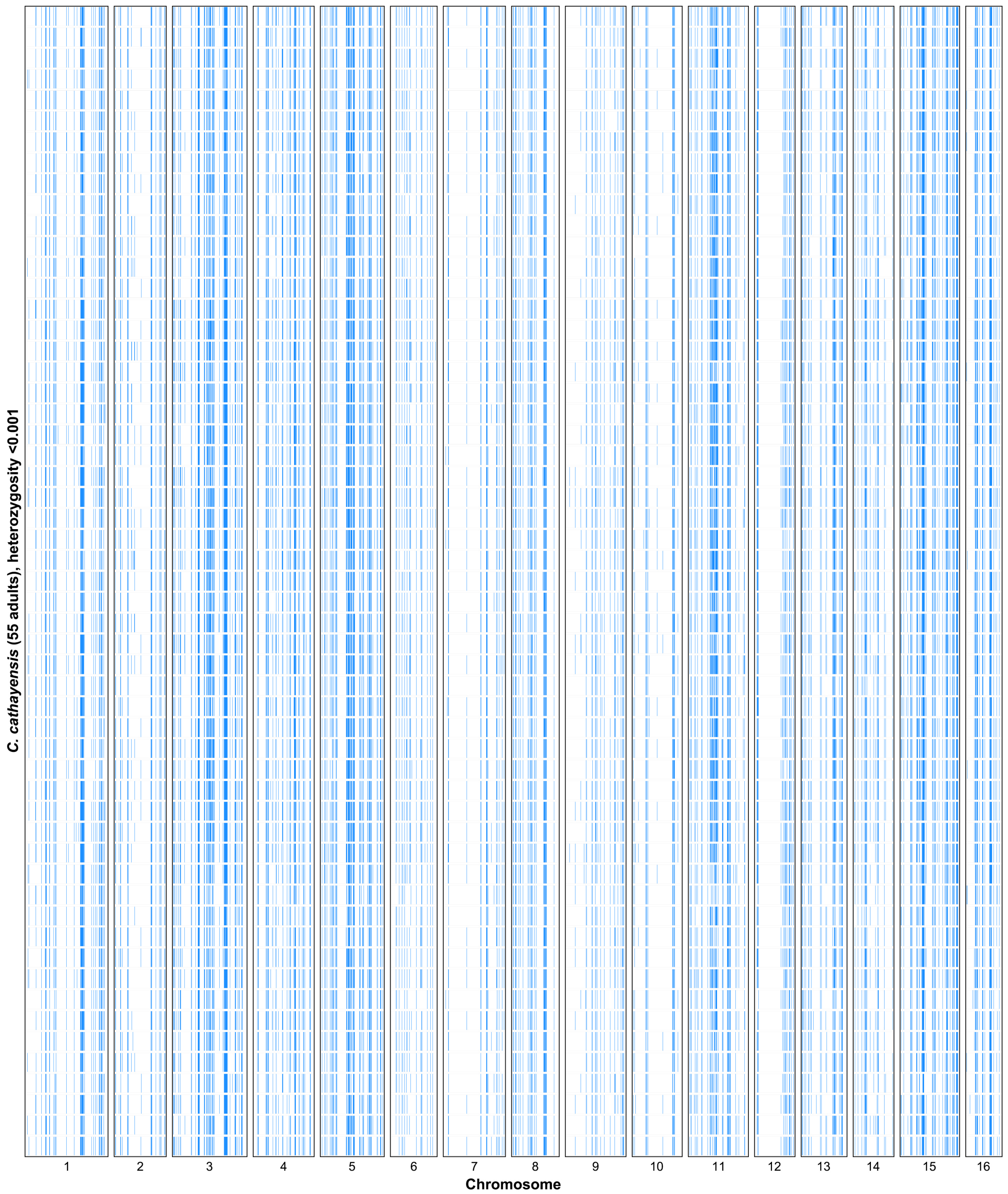

Supplementary Figure 17. Distribution of loss of heterozygosity (LOH; heterozygosity <0.001) across 16 chromosomes in 55 adults of the apomictic species *Carya cathayensis*.

LOH regions in each individual (one row per individual) were defined as 50-kb sliding windows with heterozygosity <0.001 and are shown as vertical blue bars. The recurrent and nearly identical genome-wide LOH patterns observed across individuals reflect the clonal nature of apomixis, and such LOH segments may reflect meiotic recombination and thus residual sexual reproduction. In addition, subsequent selfing or mating among closely related clonal lineages could generate novel LOH profiles distinct from those of the parental clones (the same below).

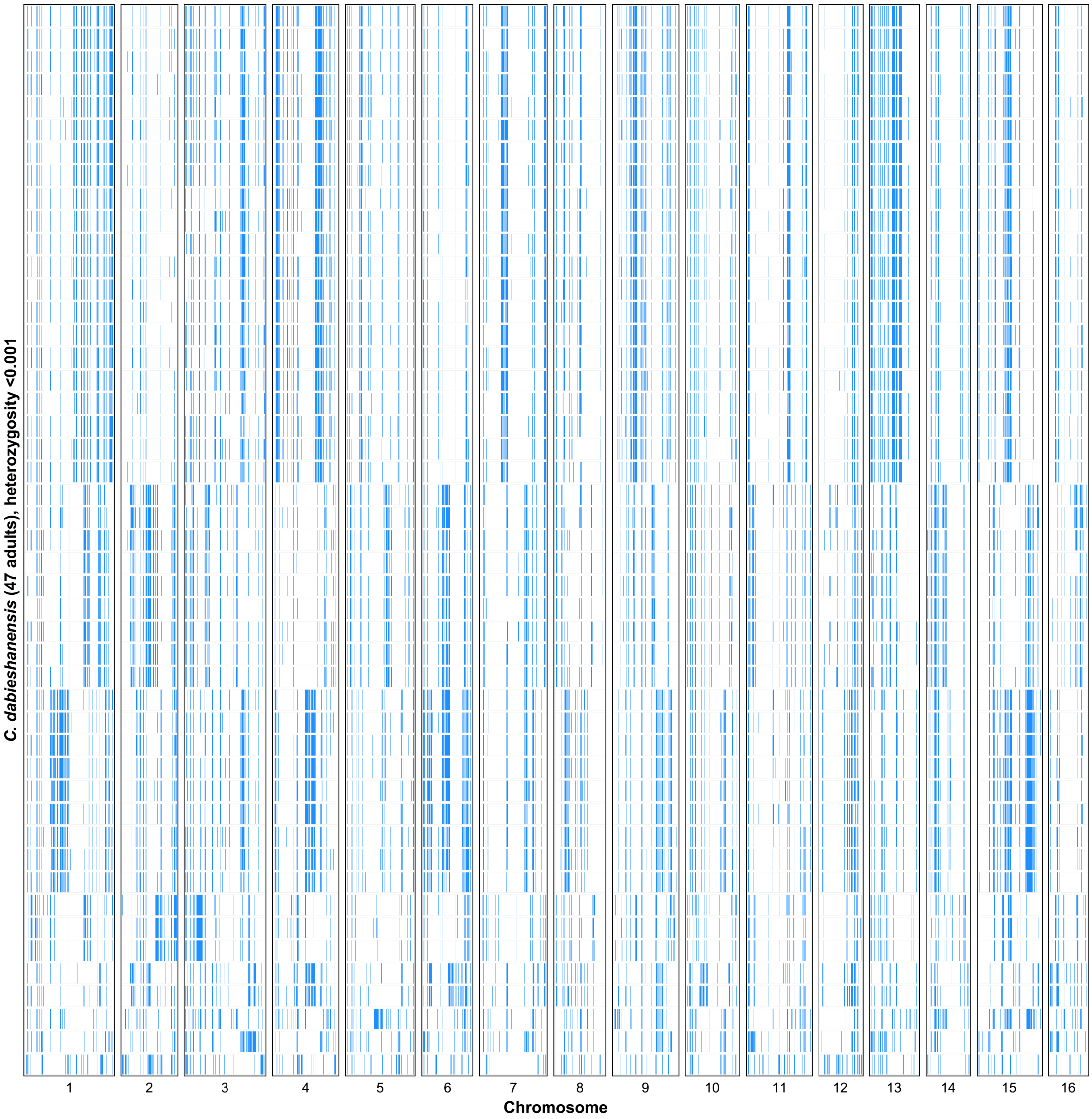

Supplementary Figure 18. Distribution of loss of heterozygosity (LOH; heterozygosity <0.001) across 16 chromosomes in 47 adults of the apomictic species *Carya dabieshanensis*.

The 47 individuals of *C. dabieshanensis* exhibited eight distinct LOH patterns, corresponding to 21, 9, 9, 3, 2, 1, 1, and 1 individual (from top to bottom), respectively. The first five groups likely represent different apomictic clonal lineages, while the last three individuals had incomplete sampling data, and their reproductive mode remains uncertain (the same below).

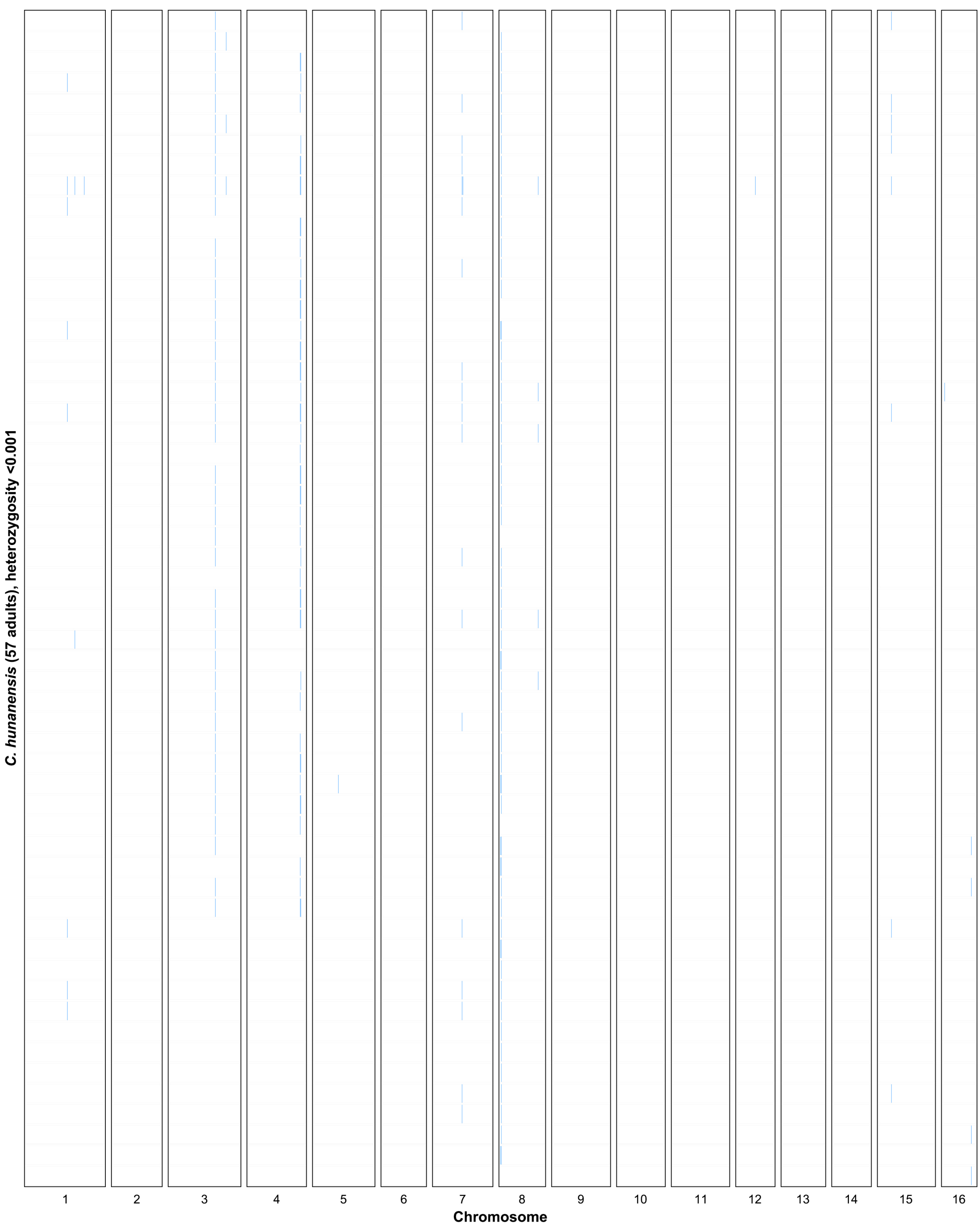

Supplementary Figure 19. Distribution of loss of heterozygosity (LOH; heterozygosity <0.001) across 16 chromosomes in 57 adults of the apomictic species *Carya hunanensis*.

Because of its hybrid origin, *C. hunanensis* exhibits elevated genomic heterozygosity, which makes LOH patterns difficult to discern. Accordingly, results under the more stringent threshold (<0.0005) are not shown.

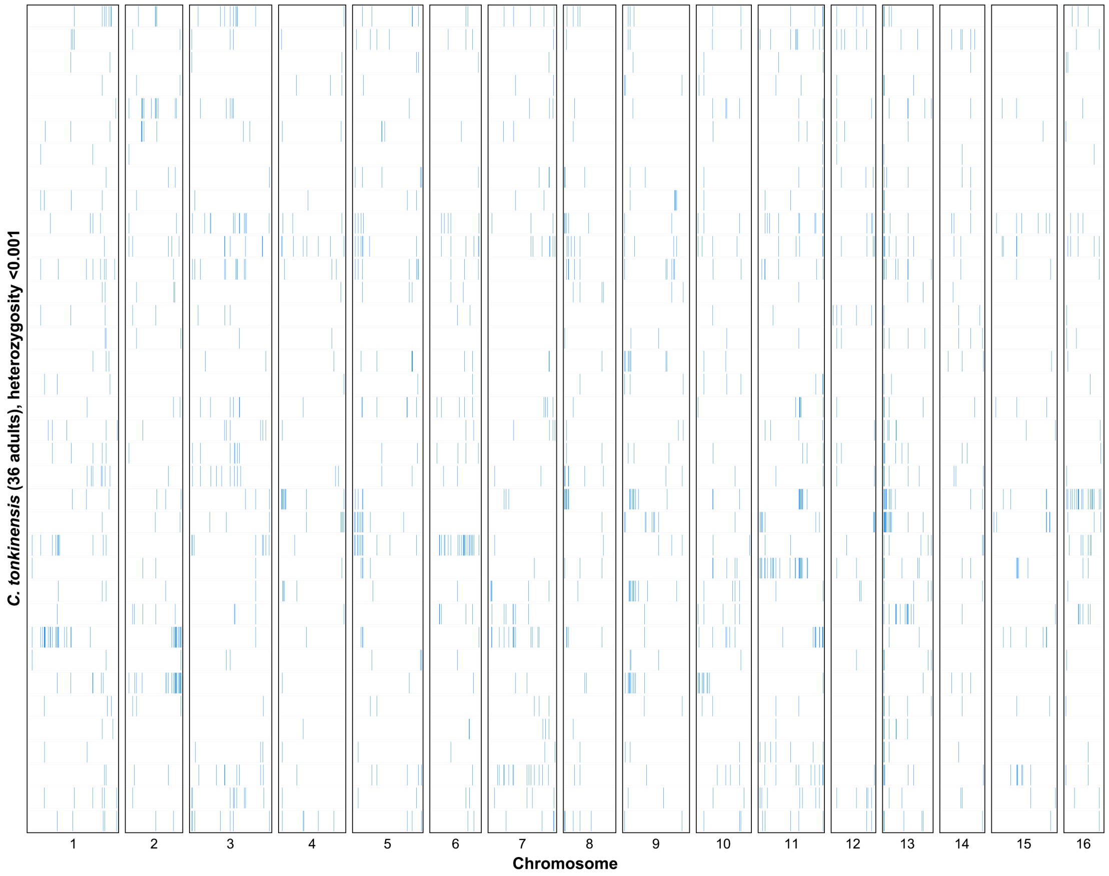

Supplementary Figure 20. Distribution of loss of heterozygosity (LOH; heterozygosity <0.001) across 16 chromosomes in 36 adults of the sexual control *Carya tonkinensis*.

As a sexually reproducing species, *C. tonkinensis* exhibits pronounced individual variation in LOH patterns across the genome, reflecting random recombination events (the same below).

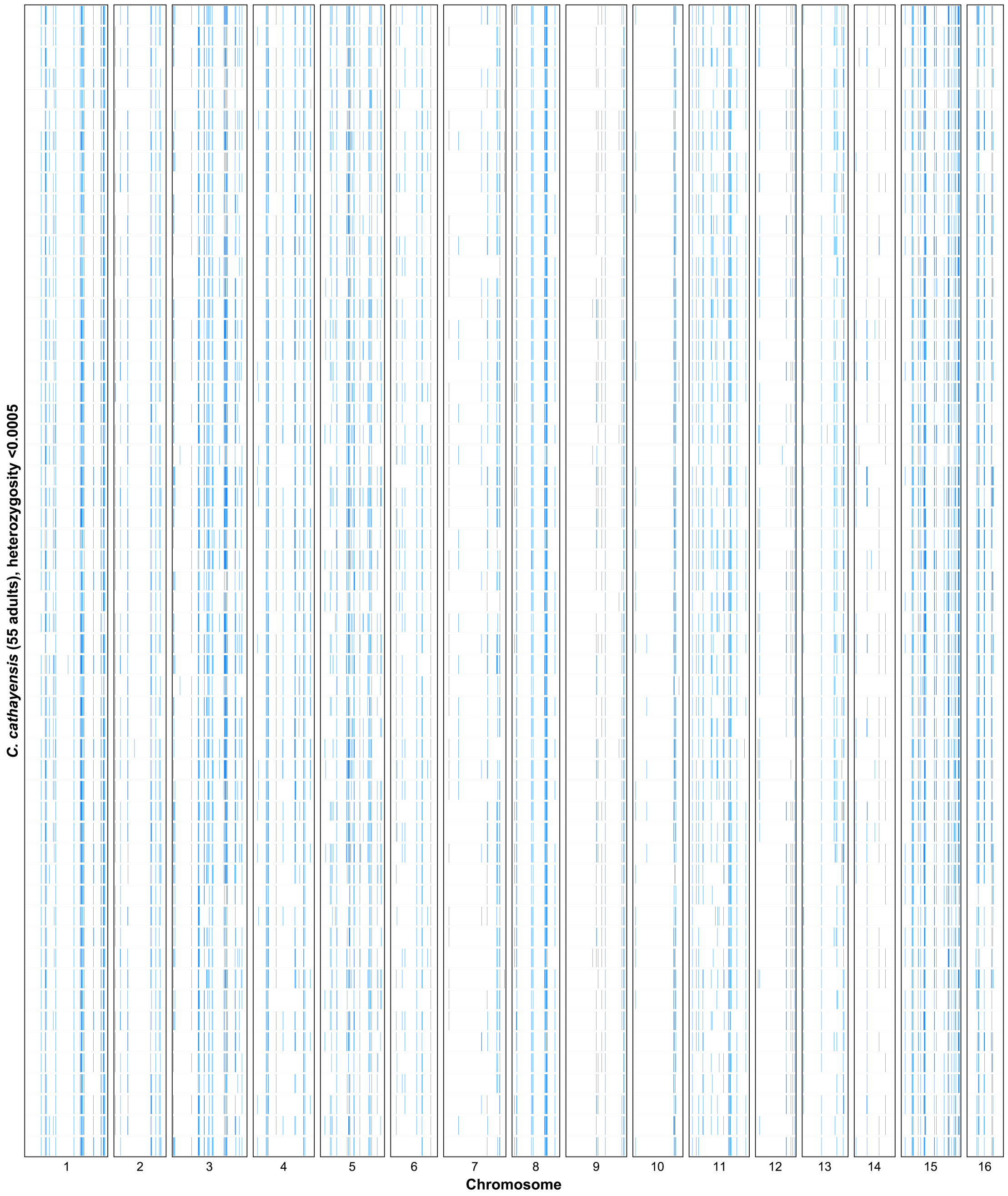

Supplementary Figure 21. Distribution of loss of heterozygosity (LOH; heterozygosity <0.0005) across 16 chromosomes in 55 adults of the apomictic species *Carya cathayensis*.

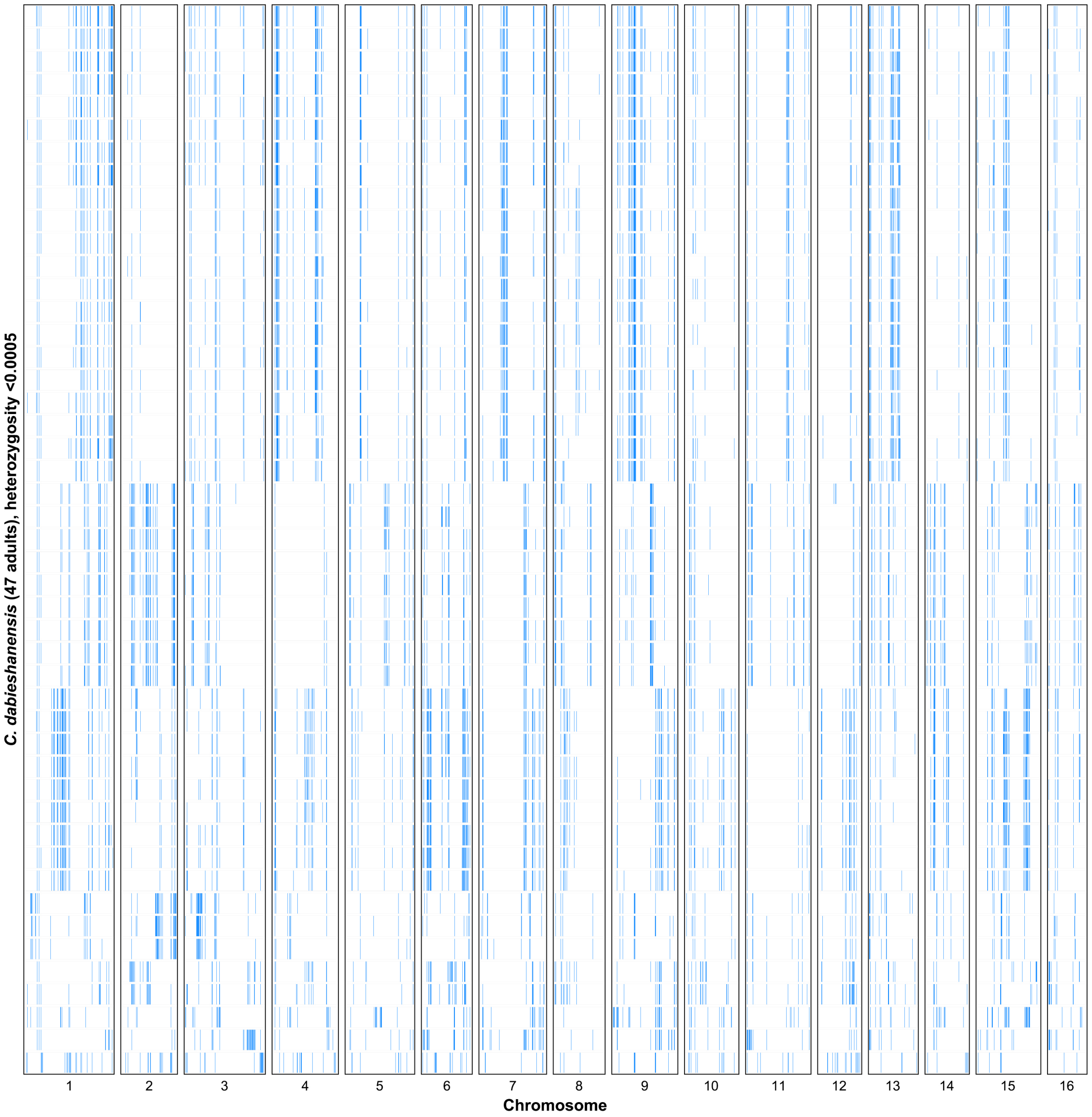

Supplementary Figure 22. Distribution of loss of heterozygosity (LOH; heterozygosity <0.0005) across 16 chromosomes in 47 adults of the apomictic species *Carya dabieshanensis*.

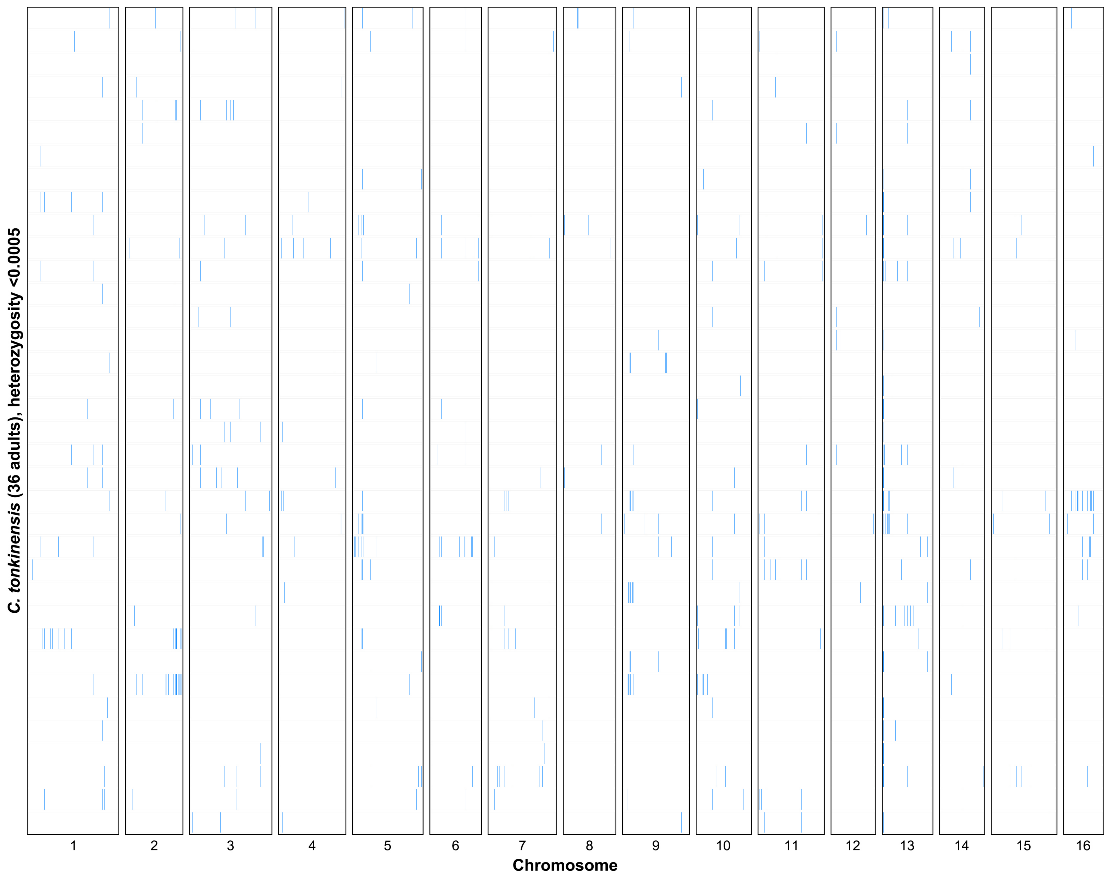

Supplementary Figure 23. Distribution of loss of heterozygosity (LOH; heterozygosity <0.0005) across 16 chromosomes in 36 adults of the sexual control *Carya tonkinensis*.

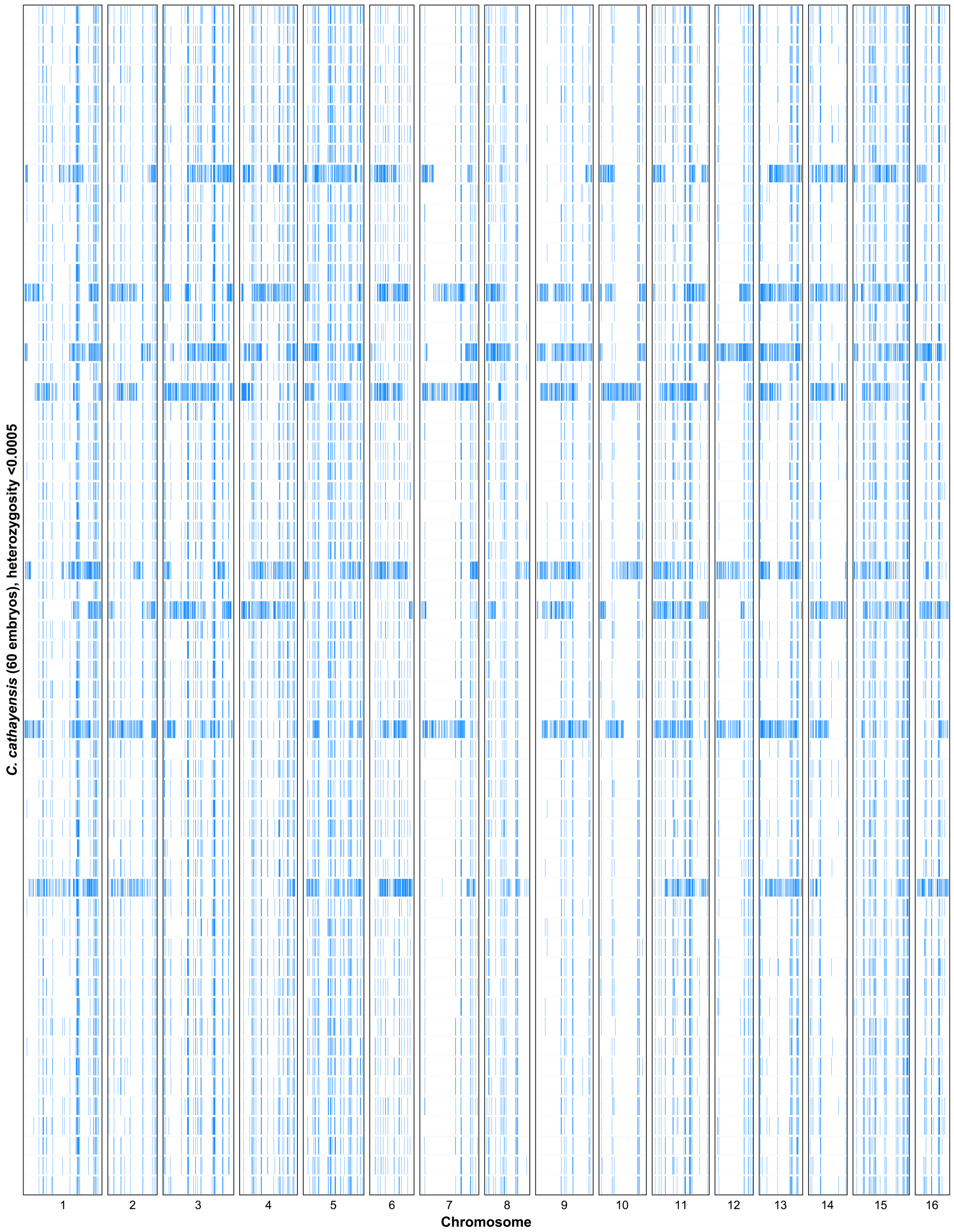

Supplementary Figure 24. Distribution of loss of heterozygosity (LOH; heterozygosity <0.0005) across 16 chromosomes in 60 mature embryos of the apomictic species *Carya cathayensis*.

In eight mature embryos, novel LOH patterns were detected that differed from the majority of apomictic embryos, indicating recombination arising from residual sexual reproduction.

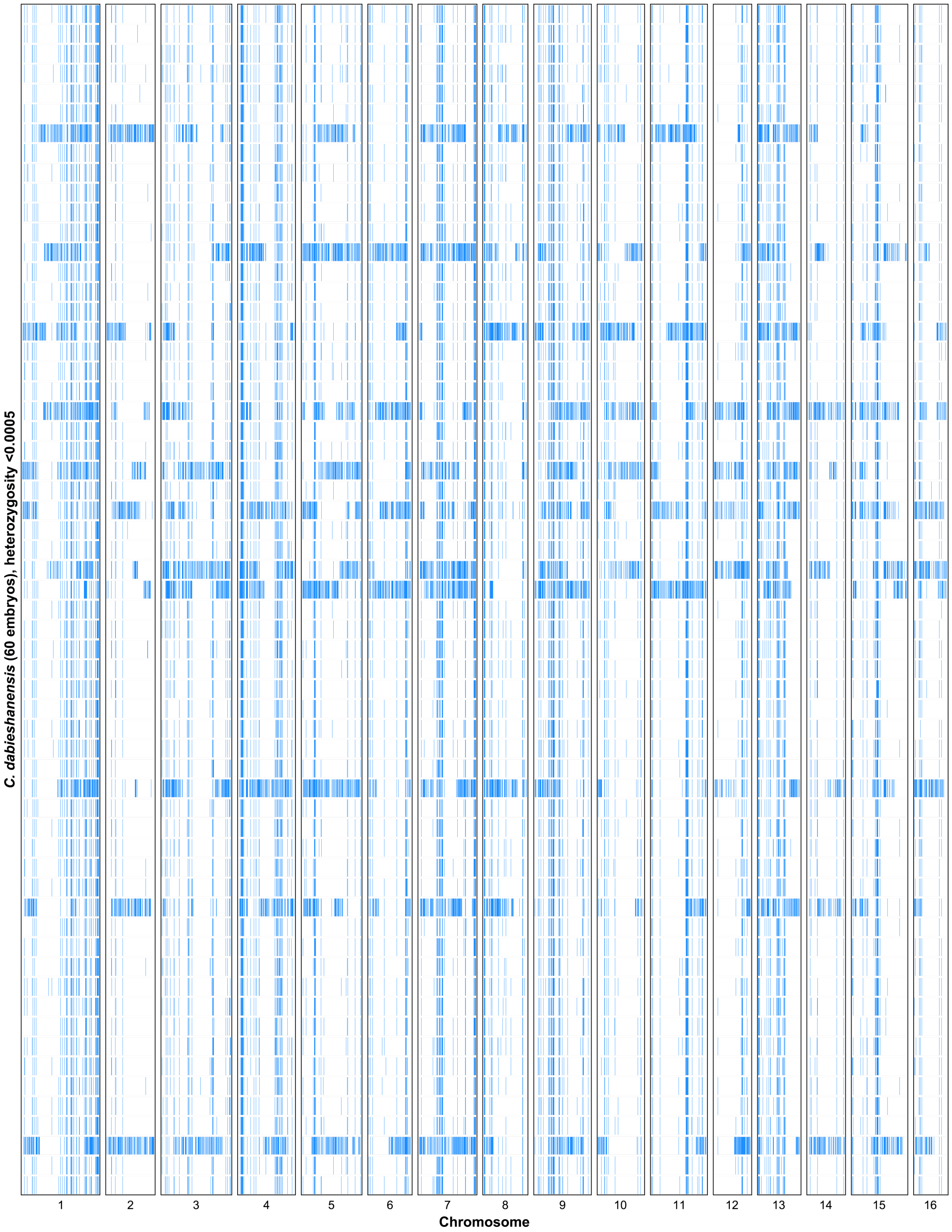

Supplementary Figure 25. Distribution of loss of heterozygosity (LOH; heterozygosity <0.0005) across 16 chromosomes in 60 mature embryos of the apomictic species *Carya dabieshanensis*.

In eleven mature embryos, novel LOH patterns were detected that differed from the majority of apomictic embryos, indicating recombination arising from residual sexual reproduction.

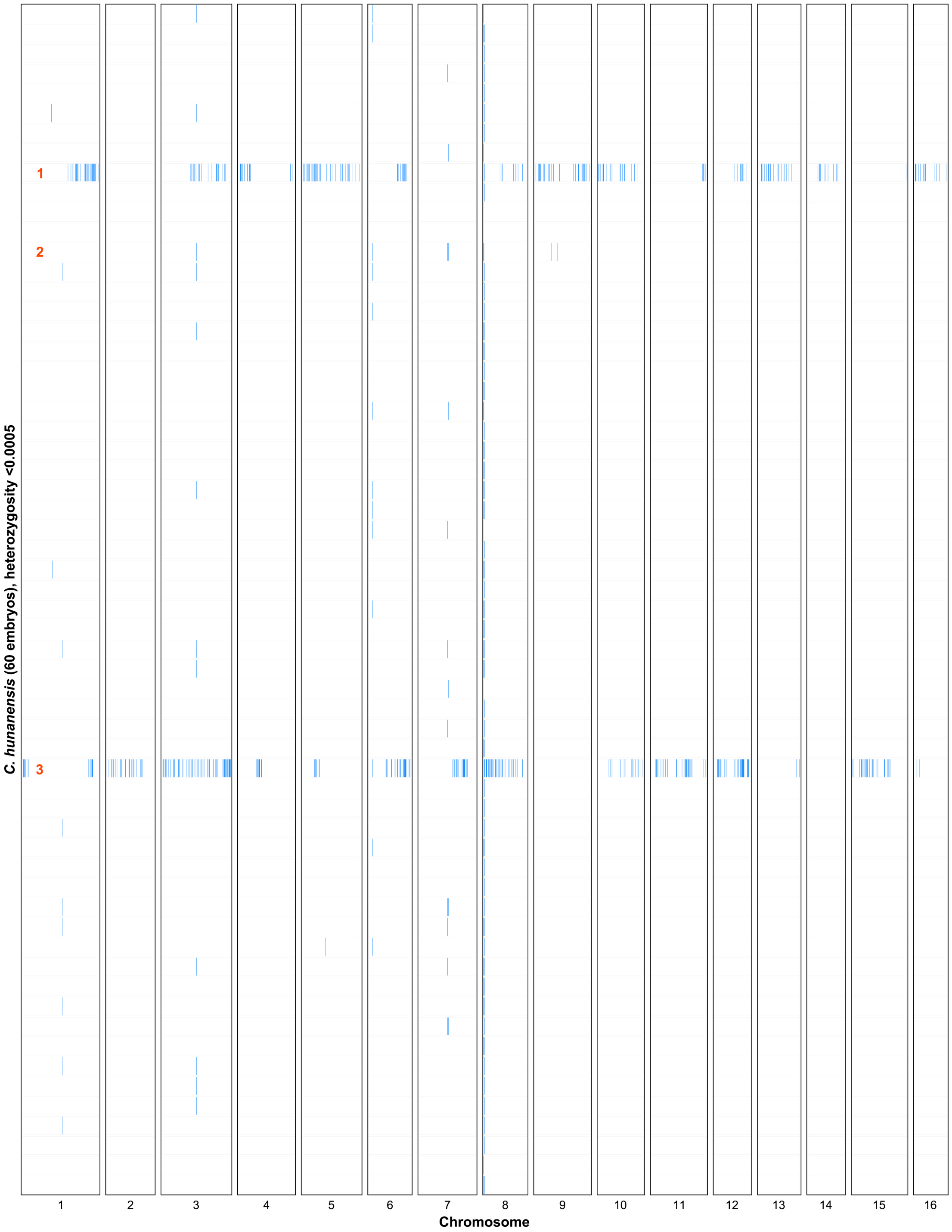

Supplementary Figure 26. Distribution of loss of heterozygosity (LOH; heterozygosity <0.0005) across 16 chromosomes in 60 mature embryos of the apomictic species *Carya hunanensis*.

In three mature embryos, novel LOH patterns were detected that differed from the majority of apomictic embryos, indicating recombination arising from residual sexual reproduction. For one of these embryos, the signal was not apparent under the more stringent 0.0005 threshold but was supported both by genome-wide heterozygosity estimates (Supplementary Fig. 31) and by LOH patterns at the 0.001 threshold (Fig. 3f).

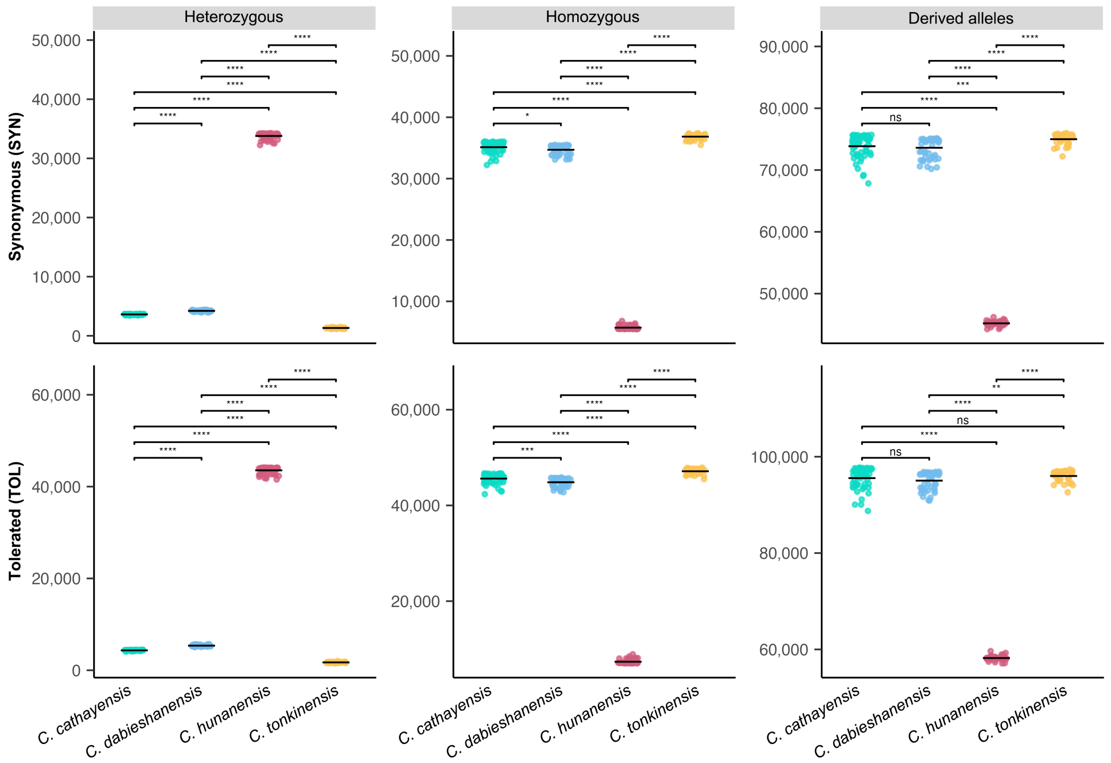

Supplementary Figure 27. Comparison of non-deleterious genetic variation in adult individuals of apomictic and sexual hickory species.

The analysis is based on 195 adult individuals from four hickory species (55 *C. cathayensis*, 47 *C. dabieshanensis*, 57 *C. hunanensis* and 36 *C. tonkinensis*). The total number of derived alleles was calculated by counting each heterozygous genotype as one and each homozygous-derived genotype as two. Results are based on SIFT4G predictions using the *C. hunanensis* haplotype E reference genome (derived from apomictic lineages). Horizontal bars denote average values. Statistical significance between species was assessed using two-sided Welch's t-tests without multiple-testing correction, and the asterisks indicate significance levels (ns, not significant; *$P < 0.05$; **$P < 0.01$; ***$P < 0.001$).

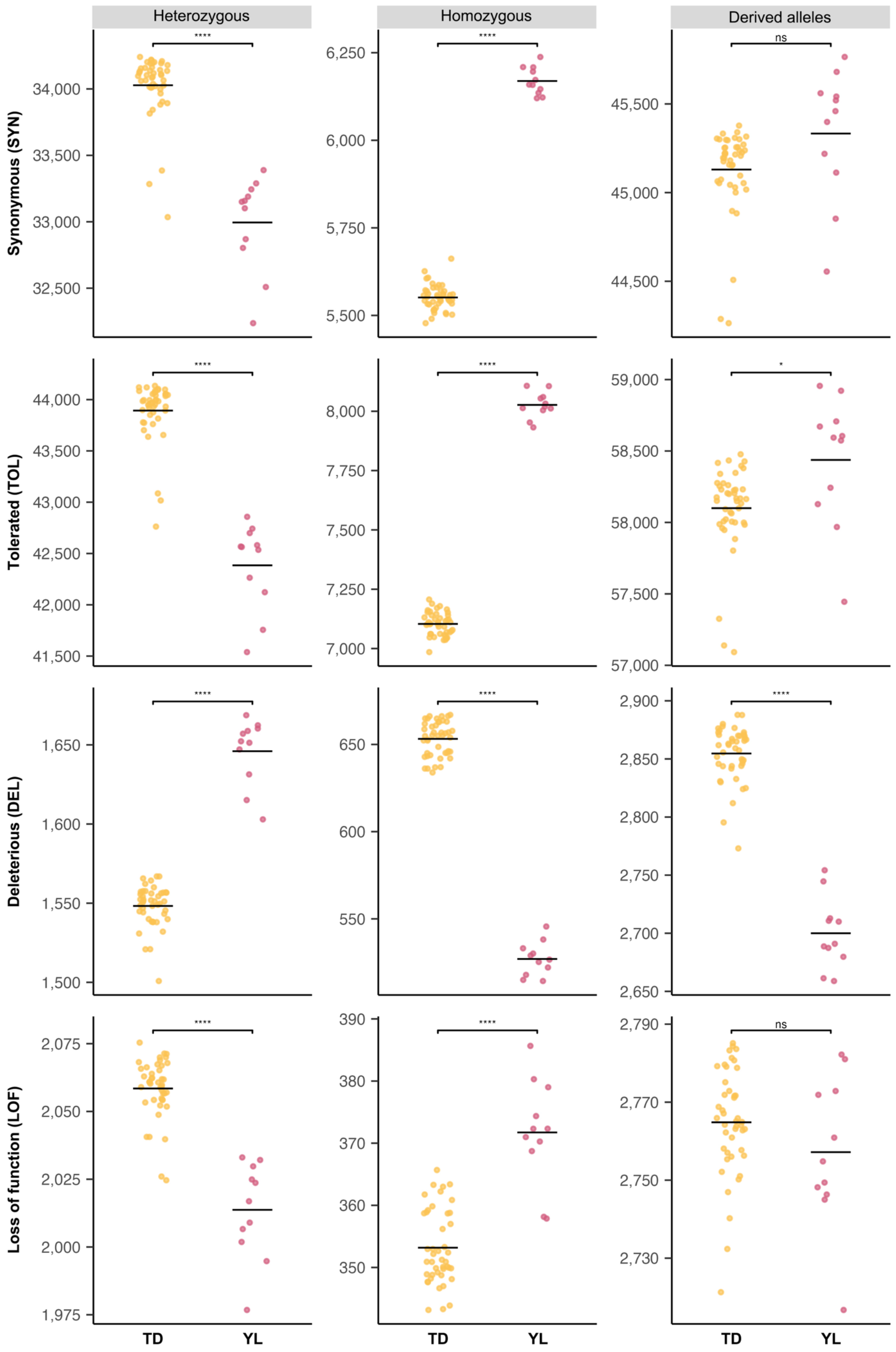

Supplementary Figure 28. Comparison of deleterious genetic variation between the two morphotypes of *Carya hunanensis*.

TD and YL represent the two morphotypes of *C. hunanensis*, with 44 and 11 individuals, respectively. The total number of derived alleles was calculated by counting each heterozygous genotype as one and each homozygous-derived genotype as two. Results are based on SIFT4G predictions using the *C. hunanensis* haplotype E reference genome (derived from apomictic lineages). Horizontal bars denote average values. Statistical significance between two morphotypes was assessed using two-sided Welch's t-tests without multiple-testing correction, and the asterisks indicate degree of significance (ns, not significant; *$P < 0.05$; **$P < 0.01$; ***$P < 0.001$).

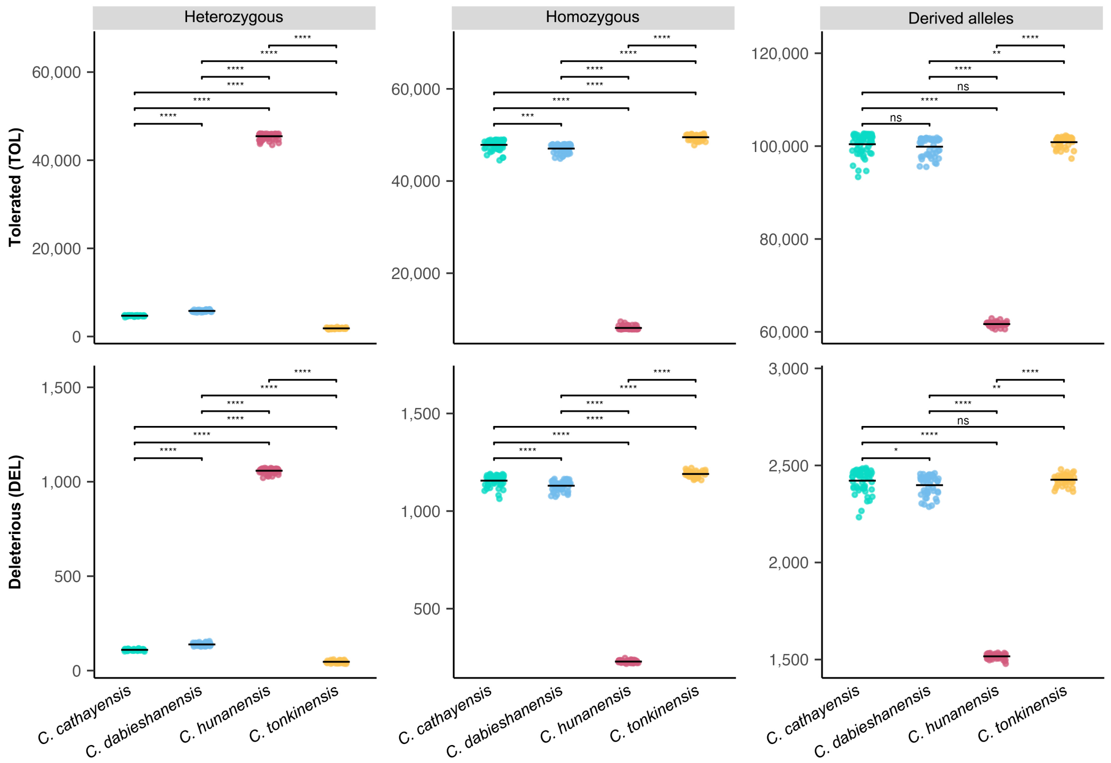

Supplementary Figure 29. Comparison of deleterious variants based on Grantham scores in adult individuals of apomictic and sexual hickory species.

The analysis is based on 195 adult individuals from four hickory species (55 *C. cathayensis*, 47 *C. dabieshanensis*, 57 *C. hunanensis* and 36 *C. tonkinensis*). Missense mutations identified using the SnpEff program were further evaluated with Grantham scores, which quantify physicochemical differences between amino acids; mutations with scores ≥150 were designated as deleterious, and those <150 as tolerated. Analyses were performed on the same dataset mapped to the *C. hunanensis* Haplotype E genome (derived from apomictic lineages). Horizontal bars denote average values. Statistical significance between species pairs was assessed using two-sided Welch's t-tests without multiple-testing correction, and the asterisks indicate degree of significance (ns, not significant; *$P < 0.05$; **$P < 0.01$; ***$P < 0.001$). See Methods in the main text for details.

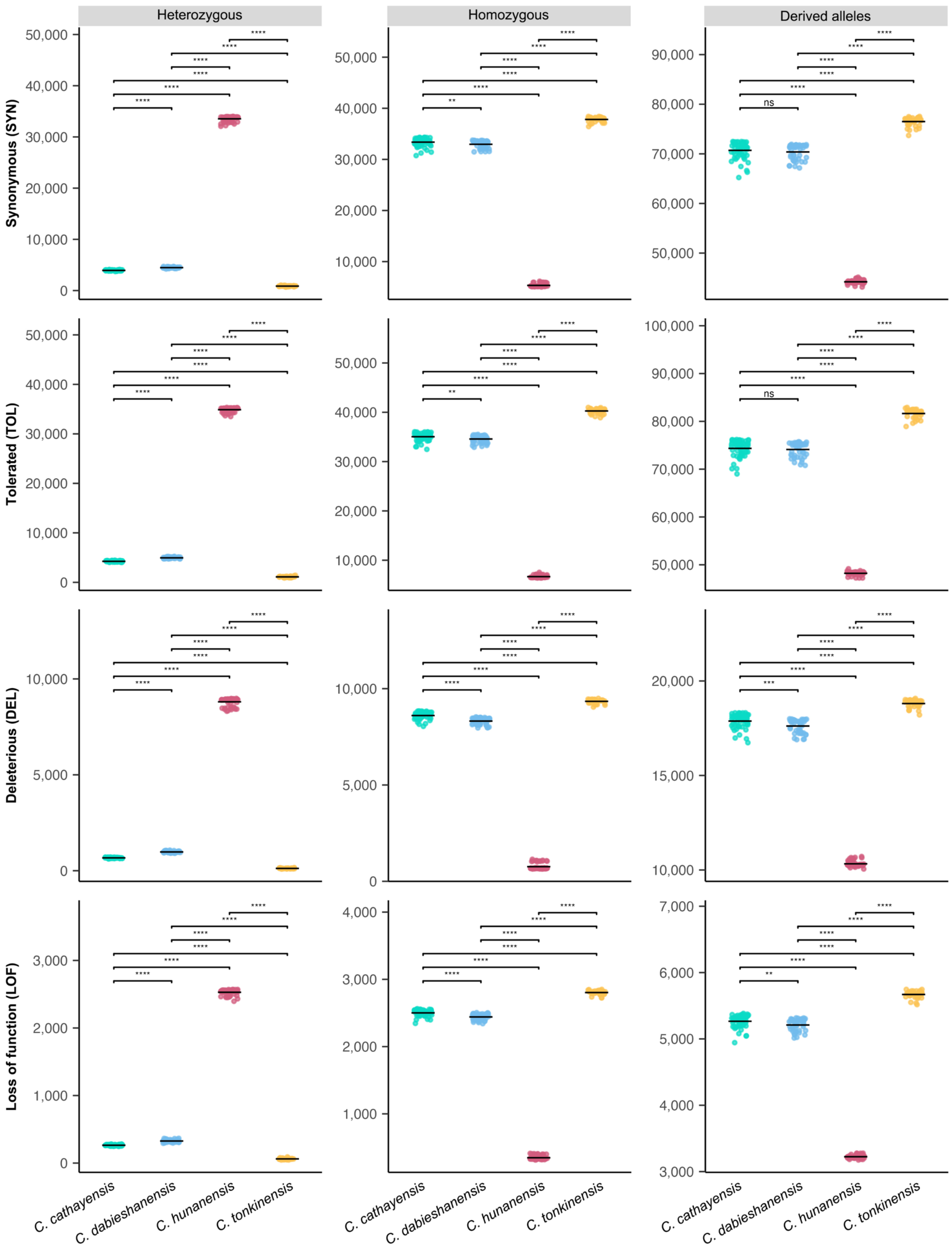

Supplementary Figure 30. Comparison of deleterious genetic variation in adult individuals of apomictic versus sexual hickory species based on SIFT4G predictions with the *Carya hunanensis* haplotype W reference genome.

The analysis is based on 195 adult individuals from four hickory species (55 *C. cathayensis*, 47 *C. dabieshanensis*, 57 *C. hunanensis* and 36 *C. tonkinensis*), and performed using the haplotype W reference genome of *C. hunanensis* (derived from sexual lineages). The total number of derived alleles was calculated by counting each heterozygous genotype as one and each homozygous-derived genotype as two. Horizontal bars denote average values. Statistical significance between species pairs was assessed using two-sided Welch's t-tests without multiple-testing correction, and

the asterisks indicate degree of significance (ns, not significant; $*P < 0.05$; $**P < 0.01$; $***P < 0.001$). See Methods in the main text for details.

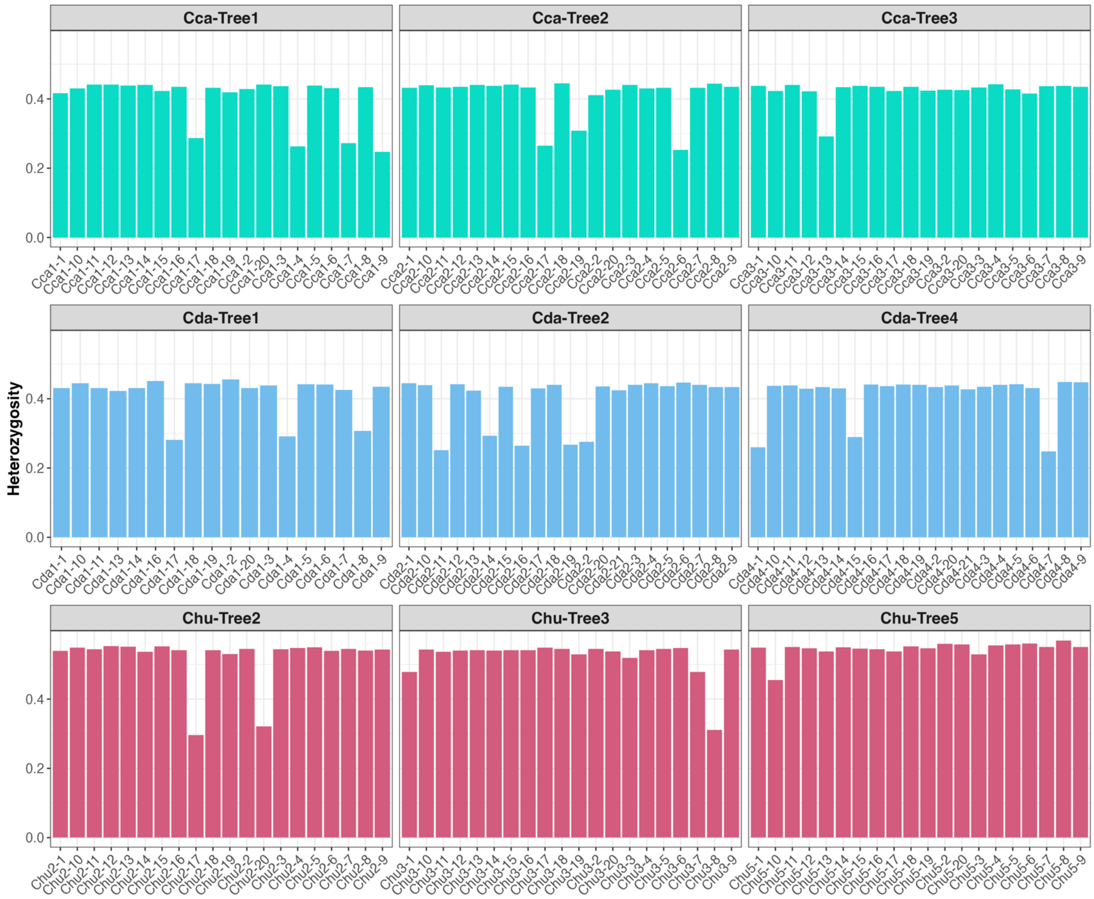

Supplementary Figure 31. Genome-wide heterozygosity of mature embryos from three apomictic hickory species.

Embryos with significantly reduced heterozygosity correspond to individuals showing recombination evidence in the loss of heterozygosity (LOH) analysis. Cca-Tree1, Cca-Tree2, and Cca-Tree3 for *Carya cathayensis*; Cda-Tree1, Cda-Tree2, and Cda-Tree4 for *C. dabieshanensis*; and Chu-Tree2, Chu-Tree3, and Chu-Tree5 for *C. hunanensis*. A total of 18–21 embryos were collected per tree. Heterozygosity was estimated for each embryo as the proportion of heterozygous genotypes (0/1) among all non-missing genotypes (0/0, 0/1, 1/1), excluding missing calls (‘./.’). This measure reflects heterozygosity on the discovered SNP panel and is appropriate for relative comparisons within this dataset.

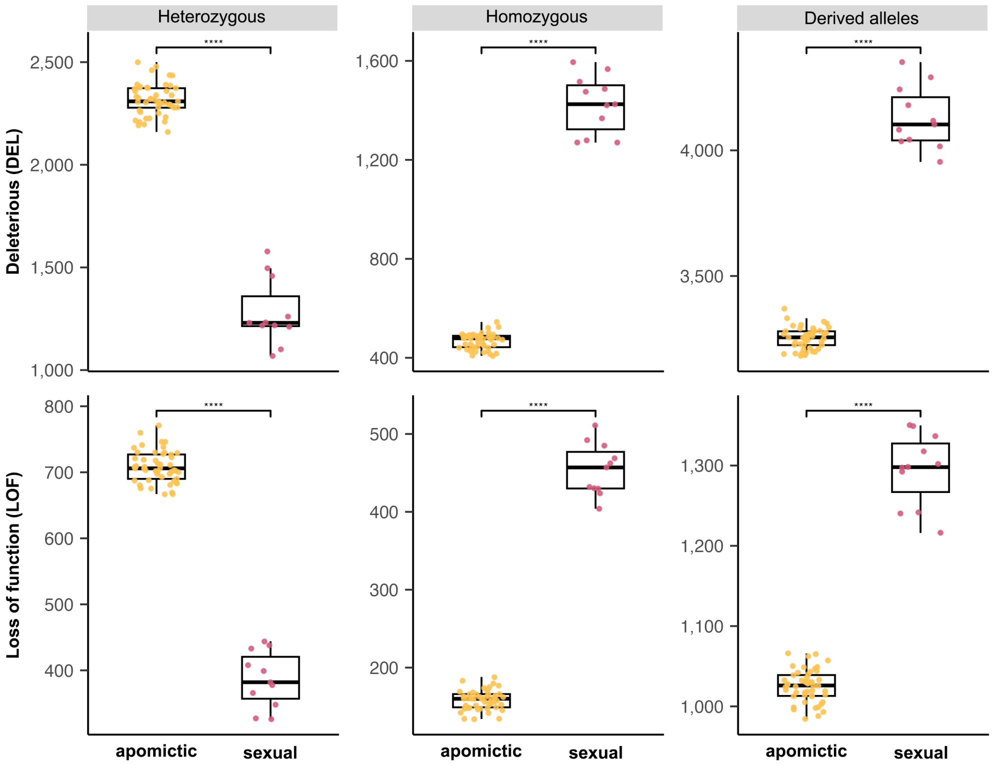

Supplementary Figure 32. Comparison of deleterious mutation loads between 49 apomictic and 11 sexual mature embryos of *Carya dabieshanensis*.

The total number of derived alleles was calculated by counting each heterozygous genotype as one and each homozygous-derived genotype as two. Box plots summarize the distribution across individuals: the center line indicates the median, the box bounds correspond to the 25th and 75th percentiles, and whiskers extend to 1.5× the interquartile range. Statistical significance between two reproductive types was assessed using two-sided Welch's t-tests without multiple-testing correction, and the asterisks indicate degree of significance (ns, not significant; *$P < 0.05$; **$P < 0.01$; ***$P < 0.001$).

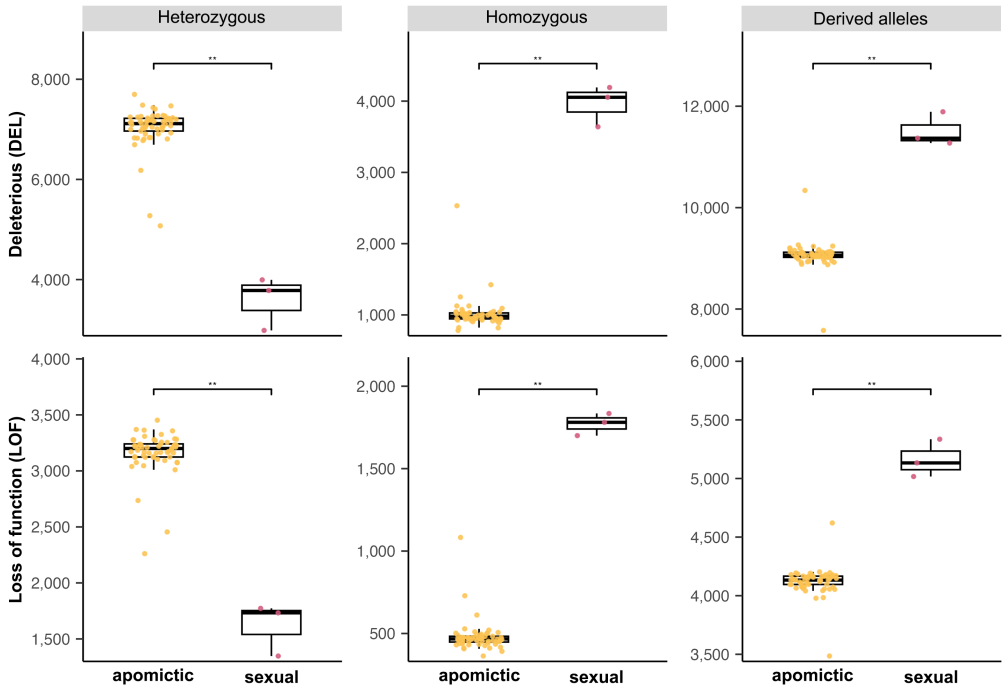

Supplementary Figure 33. Comparison of deleterious mutation loads between 57 apomictic and 3 sexual mature embryos of *Carya hunanensis*.

The total number of derived alleles was calculated by counting each heterozygous genotype as one and each homozygous-derived genotype as two. Box plots summarize the distribution across individuals: the center line indicates the median, the box bounds correspond to the 25th and 75th percentiles, and whiskers extend to 1.5× the interquartile range. Statistical significance between the two reproductive types was assessed using a two-sided Wilcoxon rank-sum test (W = 171 or 0, $P \approx 0.0039$ across categories). Effect size estimation with Cliff's delta (±1, large effect) indicated complete separation between groups. Asterisks indicate degree of significance (ns, not significant; *$P < 0.05$; **$P < 0.01$; ***$P < 0.001$).

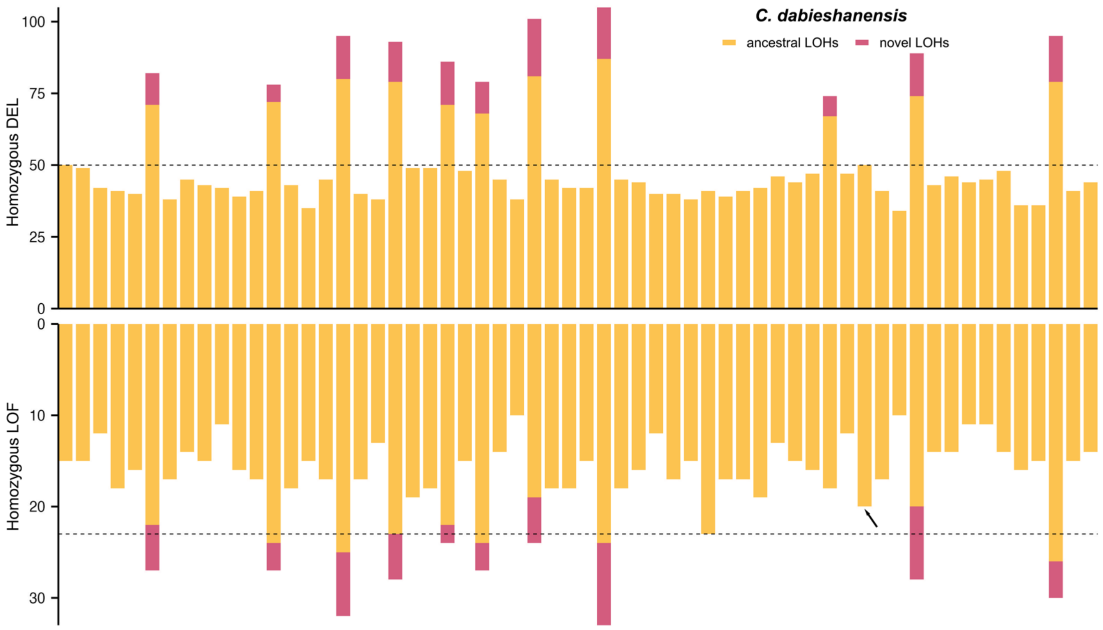

Supplementary Figure 34. Numbers of homozygous DEL and LOF mutations in ancestral LOH regions versus novel LOH regions in *Carya dabieshanensis*.

Orange bars represent ancestral LOH regions shared across all 60 embryos, while red bars indicate novel LOH regions arising via recombination in the 11 sexual embryos. The dashed line indicates the maximum number of homozygous deleterious mutations observed in apomictic embryos, and the arrow highlights the absence of LOF mutation accumulation in novel LOH regions of sexual embryos.

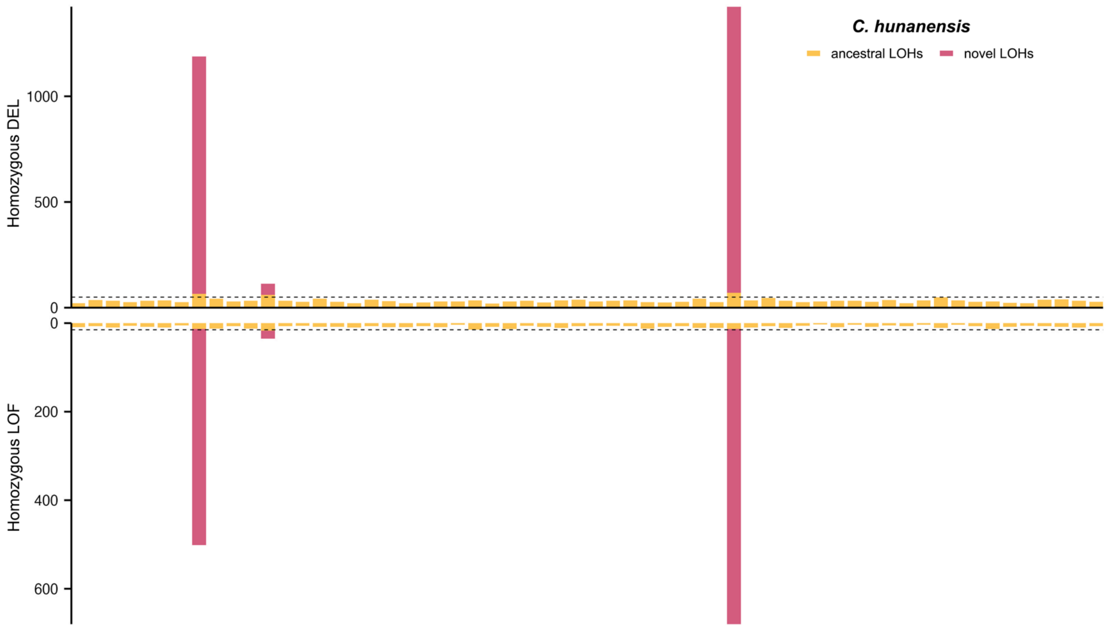

Supplementary Figure 35. Numbers of homozygous DEL and LOF mutations in ancestral LOH regions versus novel LOH regions in *Carya hunanensis*.

Orange bars represent ancestral LOH regions shared across all 60 embryos, while red bars indicate novel LOH regions arising via recombination in the 3 sexual embryos. The dashed line indicates the maximum number of homozygous deleterious mutations observed in apomictic embryos.

Supplementary Table 1. Assembly and annotation features of *Carya hunanensis* and its haplotype-resolved genomes

Genome Statistics	*C. hunanensis*	*C. hunanensis* E	*C. hunanensis* W
Ploidy level	Diploid	Haplotype	Haplotype
Illumina short reads (Gb)	31.27	-	-
PacBio single-molecule long reads (Gb)	25.07	-	-
Hi-C interaction reads (Gb)	96.93	-	-
Estimated genome size (Mb)	701.66	-	-
Assembled genome size (Mb)	1356.78	671.52	673.12
No. of scaffolds (>10kb)	705	207	302
N_{50} length (contig) (Mb)	11.16	-	-
N_{50} length (scaffold) (Mb)	43.81	44.14	43.05
L_{50} (scaffold)	14	7	7
Longest scaffold (Mb)	59.63	59.63	58.35
Chromosome anchor percent (%)	97.13	97.6	96.66
GC content (%)	36.63	36.51	36.6
Repetitive sequences (%)	50.33	-	-
Predicted protein-coding genes	78,799	38,635	39,070

Supplementary Table 2. Functional annotation of the predicted genes models for genome of *Carya hunanensis*

Annotated database	Annotated number	Annotated ratio (%)
GO_Annotation	58,252	73.92
KEGG_Annotation	56,291	71.44
KOG_Annotation	40,956	51.98
Pfam_Annotation	62,738	79.62
Swissprot_Annotation	57,713	73.24
TrEMBL_Annotation	75,576	95.91
eggNOG_Annotation	62,695	79.56
nr_Annotation	75,443	95.74
All_Annotated	75,853	96.26

Supplementary Table 3. Statistics of polyembryony occurrence in mature fruits of four hickory species after germination

Seedings	*C. cathayensis*		*C. dabieshanensis*		*C. hunanensis* JZ		*C. hunanensis* YL		*C. tonkinensis*	
	**Number**	**Ratio**	**Number**	**Ratio**	**Number**	**Ratio**	**Number**	**Ratio**	**Number**	**Ratio**
1	71	65.74%	82	75.23%	1	0.88%	2	1.77%	116	100%
2	32	29.63%	23	21.10%	32	28.07%	33	29.20%	0	0
3	5	4.63%	4	3.67%	35	30.70%	29	25.66%	0	0
4	0	0	0	0	24	21.05%	26	23.01%	0	0
5	0	0	0	0	12	10.53%	13	11.50%	0	0
6	0	0	0	0	5	4.39%	4	3.54%	0	0
7	0	0	0	0	3	2.63%	3	2.65%	0	0
8	0	0	0	0	2	1.75%	3	2.65%	0	0

Note: Samples were collected from six trees per species, with 20 fruits per tree. Germination was monitored every two days, recording the number of seeds that germinated until no further germination occurred after 30 days.

References

1. Gerlach WL, Bedbrook JR. Cloning and characterization of ribosomal RNA genes from wheat and barley. *Nucleic Acids Res.* **7**, 1869–1885 (1979).
2. Gerlach WL, Dyer TA. Sequence organization of the repeating units in the nucleus of wheat which contain 5S rRNA genes. *Nucleic Acids Res.* **8**, 4851–4865 (1980).
3. Lv F, Yang F, Zhang R. Karyotypes of three *Carya* Nutt. species (in Chinese). *J. Cent. South For. Univ.* **22**, 47–49 (2002).
4. Xu C*, et al.* Meiosis of pollen mother cell and karyotype of *Carya cathayensis* (in Chinese). *Sci. Silvae Sin.* **53**, 77–84 (2017).
5. Bolger AM, Lohse M, Usadel B. Trimmomatic: a flexible trimmer for Illumina sequence data. *Bioinformatics* **30**, 2114–2120 (2014).
6. Xie T*, et al.* De novo plant genome assembly based on chromatin interactions: a case study of *Arabidopsis thaliana*. *Mol. Plant* **8**, 489–492 (2015).
7. Servant N*, et al.* HiC-Pro: an optimized and flexible pipeline for Hi-C data processing. *Genome Biol.* **16**, 259 (2015).
8. Marcais G, Kingsford C. A fast, lock-free approach for efficient parallel counting of occurrences of *k*-mers. *Bioinformatics* **27**, 764–770 (2011).
9. Ranallo-Benavidez TR, Jaron KS, Schatz MC. GenomeScope 2.0 and Smudgeplot for reference-free profiling of polyploid genomes. *Nat. Commun.* **11**, 1432 (2020).
10. Cheng H, Concepcion GT, Feng X, Zhang H, Li H. Haplotype-resolved de novo assembly using phased assembly graphs with hifiasm. *Nat. Methods* **18**, 170–175 (2021).
11. Li H. Aligning sequence reads, clone sequences and assembly contigs with BWA-MEM. *arXiv preprint*, arXiv:1303.3997v1302 (2013).
12. Burton JN, Adey A, Patwardhan RP, Qiu R, Kitzman JO, Shendure J. Chromosome-scale scaffolding of de novo genome assemblies based on chromatin interactions. *Nat. Biotechnol.* **31**, 1119–1125 (2013).
13. Zhang WP*, et al.* Uncovering ghost introgression through genomic analysis of a distinct eastern Asian hickory species. *Plant J.* **119**, 1386–1399 (2024).
14. Jia KH*, et al.* SubPhaser: a robust allopolyploid subgenome phasing method based on subgenome-specific k-mers. *New Phytol.* **235**, 801–809 (2022).
15. Li H, Durbin R. Fast and accurate short read alignment with Burrows-Wheeler transform. *Bioinformatics* **25**, 1754–1760 (2009).
16. Simao FA, Waterhouse RM, Ioannidis P, Kriventseva EV, Zdobnov EM. BUSCO: assessing genome assembly and annotation completeness with single-copy orthologs. *Bioinformatics* **31**, 3210–3212 (2015).
17. Stanke M, Schoffmann O, Morgenstern B, Waack S. Gene prediction in eukaryotes with a generalized hidden Markov model that uses hints from external sources. *BMC Bioinf.* **7**, 62 (2006).
18. Korf I. Gene finding in novel genomes. *BMC Bioinf.* **5**, 59 (2004).

19. Keilwagen J, Hartung F, Grau J. Gemoma: Homology-based gene prediction utilizing intron position conservation and rna-seq data. *Methods Mol. Biol.* **1962**, 161–177 (2019).
20. Kim D, Langmead B, Salzberg SL. HISAT: a fast spliced aligner with low memory requirements. *Nat. Methods* **12**, 357–360 (2015).
21. Pertea M, Kim D, Pertea GM, Leek JT, Salzberg SL. Transcript-level expression analysis of RNA-seq experiments with HISAT, StringTie and Ballgown. *Nat. Protoc.* **11**, 1650–1667 (2016).
22. Tang S, Lomsadze A, Borodovsky M. Identification of protein coding regions in RNA transcripts. *Nucleic Acids Res.* **43**, e78 (2015).
23. Haas BJ*, et al.* Improving the *Arabidopsis* genome annotation using maximal transcript alignment assemblies. *Nucleic Acids Res.* **31**, 5654–5666 (2003).
24. Grabherr MG*, et al.* Full-length transcriptome assembly from RNA-Seq data without a reference genome. *Nat Biotechnol* **29**, 644-652 (2011).
25. Haas BJ*, et al.* Automated eukaryotic gene structure annotation using EVidenceModeler and the program to assemble spliced alignments. *Genome Biol.* **9**, R7 (2008).
26. Flynn JM*, et al.* RepeatModeler2 for automated genomic discovery of transposable element families. *Proc. Natl. Acad. Sci. U.S.A.* **117**, 9451–9457 (2020).
27. Bao Z, Eddy SR. Automated de novo identification of repeat sequence families in sequenced genomes. *Genome Res.* **12**, 1269–1276 (2002).
28. Price AL, Jones NC, Pevzner PA. De novo identification of repeat families in large genomes. *Bioinformatics* **21**, i351–i358 (2005).
29. Ellinghaus D, Kurtz S, Willhoeft U. LTRharvest, an efficient and flexible software for de novo detection of LTR retrotransposons. *BMC Bioinf.* **9**, 18 (2008).
30. Xu Z, Wang H. LTR_FINDER: an efficient tool for the prediction of full-length LTR retrotransposons. *Nucleic Acids Res.* **35**, W265–W268 (2007).
31. Ou S, Jiang N. LTR_retriever: a highly accurate and sensitive program for identification of long terminal repeat retrotransposons. *Plant Physiol.* **176**, 1410–1422 (2018).
32. Tarailo-Graovac M, Chen N. Using RepeatMasker to identify repetitive elements in genomic sequences. *Curr. Protoc. Bioinform.* **25**, 1.7.1–14.18.45 (2009).
33. She R, Chu JSC, Wang K, Pei J, Chen NS. genBlastA: Enabling BLAST to identify homologous gene sequences. *Genome Res.* **19**, 143–149 (2009).
34. Birney E, Clamp M, Durbin R. GeneWise and Genomewise. *Genome Res.* **14**, 988–995 (2004).
35. Chan PP, Lin BY, Mak AJ, Lowe TM. tRNAscan-SE 2.0: improved detection and functional classification of transfer RNA genes. *Nucleic Acids Res.* **49**, 9077–9096 (2021).
36. Kozomara A, Birgaoanu M, Griffiths-Jones S. miRBase: from microRNA sequences to function. *Nucleic Acids Res.* **47**, D155–D162 (2019).

37. Nawrocki EP, Eddy SR. Infernal 1.1: 100-fold faster RNA homology searches. *Bioinformatics* **29**, 2933–2935 (2013).
38. Gardner PP*, et al.* Rfam: updates to the RNA families database. *Nucleic Acids Res.* **37**, D136–D140 (2009).
