# Supplementary material for: Genomic consequences of residual recombination in a hybrid apomictic hickory complex
Source: Nat Commun. 2026 Feb 5;17:2376. doi: 10.1038/s41467-026-68867-6 (PMC12982824; doi:10.1038/s41467-026-68867-6)
Supplement: Supplementary file 3 — Reporting Summary [file 41467_2026_68867_MOESM3_ESM.pdf]

Corresponding author(s): Wei-Ning Bai, Martin Lascoux, Da-Yong Zhang, Ming Kang

Last updated by author(s): 1 Dec, 2025

Reporting Summary

Nature Portfolio wishes to improve the reproducibility of the work that we publish. This form provides structure for consistency and transparency in reporting. For further information on Nature Portfolio policies, see our Editorial Policies and the Editorial Policy Checklist.

Statistics

For all statistical analyses, confirm that the following items are present in the figure legend, table legend, main text, or Methods section.

n/a	Confirmed	
☐	☒	The exact sample size (*n*) for each experimental group/condition, given as a discrete number and unit of measurement
☐	☒	A statement on whether measurements were taken from distinct samples or whether the same sample was measured repeatedly
☐	☒	The statistical test(s) used AND whether they are one- or two-sided<br>*Only common tests should be described solely by name; describe more complex techniques in the Methods section.*
☒	☐	A description of all covariates tested
☒	☐	A description of any assumptions or corrections, such as tests of normality and adjustment for multiple comparisons
☐	☒	A full description of the statistical parameters including central tendency (e.g. means) or other basic estimates (e.g. regression coefficient) AND variation (e.g. standard deviation) or associated estimates of uncertainty (e.g. confidence intervals)
☐	☒	For null hypothesis testing, the test statistic (e.g. *F*, *t*, *r*) with confidence intervals, effect sizes, degrees of freedom and *P* value noted<br>*Give P values as exact values whenever suitable.*
☒	☐	For Bayesian analysis, information on the choice of priors and Markov chain Monte Carlo settings
☒	☐	For hierarchical and complex designs, identification of the appropriate level for tests and full reporting of outcomes
☐	☒	Estimates of effect sizes (e.g. Cohen's *d*, Pearson's *r*), indicating how they were calculated

Our web collection on statistics for biologists contains articles on many of the points above.

Software and code

Policy information about availability of computer code

Data collection	No software or custom code was used for data collection
Data analysis	Illumina, Pacbio and Hi-C sequencing methods; Jellyfish v2.3, Genomescope v2.0, LACHESIS, BWA v0.7.12, BUSCO v5.2.2, SubPhaser, Trimmomatic v0.38, SAMtools v0.1.19, SENTIEON v202112.05, PopLDdecay, ADMIXTURE, SNPRelate v1.34.1, GATK v.4.4.0.0, pixy, HyDe, BCFtools v.1.15, vcfutils.pl, IQ-TREE v. 2.1.3, DnaSP v6, PopART v. 1.7, KING v.2.1, R, SnpEff v.5.0, SIFT 4G, Est-SFS, Tbtools.<br>The custom scripts used for the LOH (loss of heterzygosity) detect analyses in this study are available for download from https://github.com/Hickory01/Apomixis-in-Hickory-Species.git

For manuscripts utilizing custom algorithms or software that are central to the research but not yet described in published literature, software must be made available to editors and reviewers. We strongly encourage code deposition in a community repository (e.g. GitHub). See the Nature Portfolio guidelines for submitting code & software for further information.

Data

Policy information about availability of data

All manuscripts must include a data availability statement. This statement should provide the following information, where applicable:

- Accession codes, unique identifiers, or web links for publicly available datasets
- A description of any restrictions on data availability
- For clinical datasets or third party data, please ensure that the statement adheres to our policy

The raw sequence data generated in this study have been deposited in GenBank under the accession number PRJNA356989 and in the National Genomics Data Center (NGDC) under the accession number PRJCA033579.

Research involving human participants, their data, or biological material

Policy information about studies with human participants or human data. See also policy information about sex, gender (identity/presentation), and sexual orientation and race, ethnicity and racism.

Reporting on sex and gender	*Use the terms sex (biological attribute) and gender (shaped by social and cultural circumstances) carefully in order to avoid confusing both terms. Indicate if findings apply to only one sex or gender; describe whether sex and gender were considered in study design; whether sex and/or gender was determined based on self-reporting or assigned and methods used. Provide in the source data disaggregated sex and gender data, where this information has been collected, and if consent has been obtained for sharing of individual-level data; provide overall numbers in this Reporting Summary. Please state if this information has not been collected. Report sex- and gender-based analyses where performed, justify reasons for lack of sex- and gender-based analysis.*
Reporting on race, ethnicity, or other socially relevant groupings	*Please specify the socially constructed or socially relevant categorization variable(s) used in your manuscript and explain why they were used. Please note that such variables should not be used as proxies for other socially constructed/relevant variables (for example, race or ethnicity should not be used as a proxy for socioeconomic status). Provide clear definitions of the relevant terms used, how they were provided (by the participants/respondents, the researchers, or third parties), and the method(s) used to classify people into the different categories (e.g. self-report, census or administrative data, social media data, etc.) Please provide details about how you controlled for confounding variables in your analyses.*
Population characteristics	*Describe the covariate-relevant population characteristics of the human research participants (e.g. age, genotypic information, past and current diagnosis and treatment categories). If you filled out the behavioural & social sciences study design questions and have nothing to add here, write "See above."*
Recruitment	*Describe how participants were recruited. Outline any potential self-selection bias or other biases that may be present and how these are likely to impact results.*
Ethics oversight	*Identify the organization(s) that approved the study protocol.*

Note that full information on the approval of the study protocol must also be provided in the manuscript.

Field-specific reporting

Please select the one below that is the best fit for your research. If you are not sure, read the appropriate sections before making your selection.

☐ Life sciences ☐ Behavioural & social sciences ☒ Ecological, evolutionary & environmental sciences

For a reference copy of the document with all sections, see nature.com/documents/nr-reporting-summary-flat.pdf

Ecological, evolutionary & environmental sciences study design

All studies must disclose on these points even when the disclosure is negative.

Study description	This study investigates the genomic evidence of residual sex and recombination in apomixis (a form of asexual reproduction reported in plants), and assess their potential genomic consequences for apomictic lineages in four hickory species (Carya, Juglandaceae), which together represent a hybrid apomictic complex comprising both sexual and asexual lineages. We assembled a haplotype-resolved, chromosome-level reference genome for the diploid Carya hunanensis (2n = 2x = 32), and performed whole-genome resequencing of 131 newly collected leaf samples from adult trees, along with 64 publicly available genomes, resulting in a dataset of 195 individuals representing four Carya species (C. cathayensis, C. dabieshanensis, C. hunanensis and C. tonkinensis). In addition, we generated a novel dataset by sequencing 180 mature embryos from three apomictic species (C. cathayensis, C. dabieshanensis, and C. hunanensis).
Research sample	Sampling location are reported in the Supplementary Datas 1–2.
Sampling strategy	Leaf sampling was guided by previous studies on other Juglandaceae species (e.g., Zhang et al., 2022; https://academic.oup.com/mbe/article/39/1/msab308/6409197). In total, we collected samples from six Carya species, including four forming the hybrid

	apomictic complex (C. cathayensis, C. dabieshanensis, C. hunanensis, and C. tonkinensis), along with two closely related species (C. illinoinensis and C. kweichowensis) serving as outgroups. For each adult tree, three to six young leaves were sampled, and one whole mature embryo was collected per fruit for subsequent DNA extraction and sequencing.
Data collection	Plant material for each individual listed in Supplementary Datas 1–2 was collected once in the field by the first author (Wei-Ping Zhang), and DNA sequencing was performed by commercial providers (Novogene and Biomarker Technologies).
Timing and spatial scale	Empirical data has been collected 2018-2021 across southern China.
Data exclusions	n/a
Reproducibility	DNA isolation from leaf and embryo samples, as well as the germination experiment, was conducted once in this study. Most of our genomic analyses were repeated and cross-validated using multiple complementary methods.
Randomization	n/a
Blinding	Investigators were not blinded during data collection or analysis. The methodologies employed in this study are not sensitive to human prior knowledge or subjective influence. For example, the identification of reproductive modes, population structure, and genetic load was based entirely on the biological characteristics of the species and standardized, objective genomic analyses, without human interference or bias in the outcomes.

Did the study involve field work? ☒ Yes ☐ No

Field work, collection and transport

Field conditions	Our fieldwork primarily focused on recording species identification, distribution information (including geographic coordinates, elevation), habitat characteristics, tree size, phenology, fruit production, and other taxonomic and morphological traits. Other environmental variables not directly relevant to the scope of this study were not included.
Location	The four Carya species investigated in this study were sampled from several provinces in southern China, with detailed sampling locations provided in the Supplementary Datas 1–2.
Access & import/export	No access or collection permits, nor import/export permits, were required for the collection of leaf samples from the four hickory species included in this study.
Disturbance	No habitat disturbance was cause by the leaf and fruit collection.

Reporting for specific materials, systems and methods

We require information from authors about some types of materials, experimental systems and methods used in many studies. Here, indicate whether each material, system or method listed is relevant to your study. If you are not sure if a list item applies to your research, read the appropriate section before selecting a response.

Materials & experimental systems

n/a	Involved in the study	
☒	☐	Antibodies
☒	☐	Eukaryotic cell lines
☒	☐	Palaeontology and archaeology
☒	☐	Animals and other organisms
☒	☐	Clinical data
☒	☐	Dual use research of concern
☐	☒	Plants

Methods

n/a	Involved in the study	
☒	☐	ChIP-seq
☒	☐	Flow cytometry
☒	☐	MRI-based neuroimaging

Plants

Seed stocks	All adult leaf and mature fruit (embryo) samples were collected from natural populations in the field. A permanent voucher specimen for each Carya species and individual sampled in this study has been deposited in the BNU Herbarium.
Novel plant genotypes	n/a
Authentication	n/a
