# Supplementary material for: Genomic consequences of residual recombination in a hybrid apomictic hickory complex
Source: Nat Commun. 2026 Feb 5;17:2376. doi: 10.1038/s41467-026-68867-6 (PMC12982824; doi:10.1038/s41467-026-68867-6)
Supplement: Supplementary file 4 — Descriptions of Additional Supplementary Files [file 41467_2026_68867_MOESM4_ESM.pdf]

Descriptions of Additional Supplementary Files

File Name: Supplementary Data 1

Description: Genome resequencing data from leaf samples of four hickory species associated with apomixis.

File Name: Supplementary Data 2

Description: Genome resequencing data from 180 embryo samples across three apomictic hickory species.

File Name: Supplementary Data 3

Description: Results of the species-level hybridization detection analyses using HyDe software.

File Name: Supplementary Data 4

Description: GO enrichment analysis of homozygous deleterious (DEL) and loss-of-function (LOF) mutations in adult individuals of the three apomictic hickory species.
